# Stress and interferon signalling-mediated apoptosis contributes to pleiotropic anticancer responses induced by targeting NGLY1

**DOI:** 10.1038/s41416-018-0265-9

**Published:** 2018-11-02

**Authors:** Ashwini Zolekar, Victor. J. T. Lin, Nigam M. Mishra, Yin Ying Ho, Hamed S. Hayatshahi, Abhishek Parab, Rohit Sampat, Xiaoyan Liao, Peter Hoffmann, Jin Liu, Kyle A. Emmitte, Yu-Chieh Wang

**Affiliations:** 1 Department of Pharmaceutical Sciences, UNT System College of Pharmacy, University of North Texas Health Science Center, Fort Worth, TX USA; 2 Adelaide Proteomics Centre, The University of Adelaide, Adelaide, Australia; 3 Department of Mathematics, Purdue University, West Lafayette, Indiana, USA; 4 Department of Pathology, University of California, San Diego, San Diego, CA USA; 5 Future Industries Institute, University of South Australia, Adelaide, Australia; 6 Present Address: Department of Pathology and Laboratory Medicine, University of Rochester Medical Center, Rochester, NY USA

## Abstract

**Background:**

Although NGLY1 is known as a pivotal enzyme that catalyses the deglycosylation of denatured glycoproteins, information regarding the responses of human cancer and normal cells to NGLY1 suppression is limited.

**Methods:**

We examined how NGLY1 expression affects viability, tumour growth, and responses to therapeutic agents in melanoma cells and an animal model. Molecular mechanisms contributing to NGLY1 suppression-induced anticancer responses were revealed by systems biology and chemical biology studies. Using computational and medicinal chemistry-assisted approaches, we established novel NGLY1-inhibitory small molecules.

**Results:**

Compared with normal cells, NGLY1 was upregulated in melanoma cell lines and patient tumours. NGLY1 knockdown caused melanoma cell death and tumour growth retardation. Targeting NGLY1 induced pleiotropic responses, predominantly stress signalling-associated apoptosis and cytokine surges, which synergise with the anti-melanoma activity of chemotherapy and targeted therapy agents. Pharmacological and molecular biology tools that inactivate NGLY1 elicited highly similar responses in melanoma cells. Unlike normal cells, melanoma cells presented distinct responses and high vulnerability to NGLY1 suppression.

**Conclusion:**

Our work demonstrated the significance of NGLY1 in melanoma cells, provided mechanistic insights into how NGLY1 inactivation leads to eradication of melanoma with limited impact on normal cells, and suggested that targeting NGLY1 represents a novel anti-melanoma strategy.

## Background

As a pivotal glycosidase known for catalysing the removal of glycans from *N*-glycosylated asparagine residues, NGLY1 (a.k.a. PNGase) enables the deglycosylation of denatured glycoproteins and allows proteasome-mediated protein degradation to efficiently occur.^1–5^ A TGase-superfamily (PNGase-core) domain exists in NGLY1 proteins ranging from yeast to human,^4^ suggesting the evolutionarily conserved significance of NGLY1 enzymatic activity in cells. It is known that loss of NGLY1 function in cells can cause the accumulation of aberrant proteins in the cytosol and the interruption of endoplasmic reticulum-associated protein degradation (ERAD).^1,4,5^ Therefore, NGLY1 defects are likely to affect the quality control and homeostasis of many cellular proteins, subsequently perturbing signalling pathways, cell physiology, and organ development. The studies of an *NGLY1* ortholog gene, *PNGase-like* (*Pngl*), in fruit fly and fungus also indicate that NGLY1 could be involved in the regulation of cell normality through an enzymatic activity-independent mechanism.^6,7^ Interestingly, human *NGLY1* gene mutations that result in NGLY1 deficiency, a congenital deglycosylation disorder, were recently identified.^2,5,8,9^ Many of these mutations cause premature termination of translation, leading to complete loss of NGLY1 in the patients. Until this discovery, the disease implications of NGLY1 had not been definitive.

NGLY1 deficiency causes a broad spectrum of disease phenotypes with incomplete penetrance in patients.^2,5,8,9^ Many NGLY1 deficiency-associated phenotypes are closely related to developmental delay and congenital abnormalities, suggesting the significant role and intricate regulation of this glycosidase in the normal development of human organs. Despite the recently gained knowledge about NGLY1 deficiency, there is limited information regarding the responses of human cancer cells and terminally differentiated somatic cells to NGLY1 suppression. NGLY1 is commonly expressed in many types of normal and cancer cells (www.proteinatlas.org),^10^ suggesting that NGLY1 could be essential for a variety of human cells regardless of their pathophysiological conditions. Notably, NGLY1 appears to be highly expressed in certain human cancer cells (*e.g*., melanoma and ovarian cancer), while low-to-undetectable in their normal counterpart tissues (*e.g*., skin and ovary) (www.proteinatlas.org).^10^ These observations raise an intriguing possibility that NGLY1 may be crucial for cancer development and progression. Moreover, cancer cells, at least in certain cancer types, may be particularly vulnerable to loss of NGLY1 compared with normal cells.

In this study, we systematically and mechanistically examined the significance of NGLY1 in melanoma cells and how it may be exploited as a novel anticancer target. In addition, we developed and tested novel covalent inhibitors that suppress NGLY1 activity in human cells. Our results strongly support that NGLY1 suppression in melanoma cells elicits multifaceted cancer-elimination responses, and that targeting NGLY1 and protein deglycosylation may represent a novel anticancer strategy with the opportunity for a broad therapeutic window.

## Materials and methods

### Cell Culture

Human dermal fibroblasts were cultured in DMEM (Thermo Fisher Scientific, Carlsbad, CA) containing 10% fetal bovine serum (FBS; Thermo Fisher Scientific, Carlsbad, CA) at 37°C. HEMl and HEMd (ScienCell Research Laboratories, Carlsbad, CA) cells were cultured in melanocyte medium (MelM; ScienCell Research Laboratories, Carlsbad, CA). Human melanoma cells were cultured using RPMI-1640 medium (Thermo Fisher Scientific, Carlsbad, CA) or DMEM/F12 medium (Thermo Fisher Scientific, Carlsbad, CA) containing 10% FBS. WA09 human embryonic stem cells (hESCs) were obtained from the WiCell Stem Cell Bank (WiCell Research Institute, Madison, WI). HMi-506^11^ and NGLY1Pt1i-509 hiPSCs were established using CytoTune Sendai Reprogramming Kit (Thermo Fisher Scientific, Carlsbad, CA). We followed the previously described method^12^ for culturing undifferentiated human pluripotent stem cells (hPSCs) in a feeder cell-free condition, except the use of TeSR-E8 medium (Stemcell Technologies, Vancouver, Canada) and L7 hPSC passaging solution (Lonza, Walkersville, MD) in this study. The detailed information of cells used in this study was summarised in Supplementary Table S1. The experiments using hPSCs were performed in compliance with the guidelines and approval of the institutional biosafety committee at UNTHSC. All cells were periodically tested using the MycoAlert mycoplasma detection kit (Lonza, Walkersville, MD) and free of mycoplasma.

### Melanoma Patient Samples

The RNA samples of tumour tissues from randomly selected melanoma patients were obtained from OriGene Technologies (Rockville, MD). The tissue arrays that contain 8 cases of human normal skin and 36 cases of melanoma tumours were acquired from BioChain Institute (Newark, CA).

### CRISPR-Cas9-mediated Gene Editing

For CRISPR-Cas9-mediated gene editing to knockout the expression of NGLY1 in hPSCs, we designed two *NGLY1*-targeting sgRNA sequences (sgRNA37:^5^^’^CATTCAACAGCTCCTCTGAC^3^^’^ and sgRNA39:^5^^’^GATCTGATGACTGCCCTTGA^3^^’^) using the CRISPR Design Tool (http://crispr.mit.edu/). These two sgRNA sequences were independently cloned into a lentiCRISPRv2 plasmid (Addgene, Cambridge, MA) to generate two constructs of an one-vector system for sgRNA and Cas9 expression. WA09 hESCs transduced with the sgRNA and Cas9 expression constructs were selected using puromycin and subjected to a single-cell cloning process. Using a surveyor mutation detection kit (Integrated DNA Technologies, Coralville, IA) to examine indel mutations at the editing sites followed by western blotting to test NGLY1 expression, hESCs with *NGLY1* gene mutations that lead to the ablation of NGLY1 expression were chosen and further expanded.

### Knockdown of NGLY1 and GADD153

The knockdown of NGLY1 expression in melanoma cells was achieved by the transduction of pZIP-TRE3GS lentiviral expression vectors that carry two independent shRNA sequences (Supplementary Materials and Methods; TransOMIC Technologies, Huntsville, AL). A pZIP-TRE3GS vector that carries a NT-shRNA sequence was used as the control. The expression of the shRNA sequences and an open reading frame of the ZsGreen reporter is driven by the TRE3GS doxycycline-inducible promoter. The transduced cells were selected using puromycin for a prolonged period (~4 weeks) to obtain the stable clones of cancer cells that carry inducible NT-shRNA, NGLY1-shRNA645 and NGLY1-shRNA647 sequences.

The knockdown of GADD153 expression in melanoma cells was achieved by the transduction of pZIP-hEF1a-RFP lentiviral expression vectors that carry three independent shRNA sequences (Supplementary Materials and Methods; TransOMIC Technologies, Huntsville, AL). A pZIP-hEF1a-RFP lentiviral expression vector carries a NT-shRNA sequence was used as the control. The expression of the shRNA sequences and an open reading frame of the RFP reporter is driven by the human *EF1α* gene promoter.

### Overexpression of human NGLY1

A pLenti expression vector that carries a Myc-DDK-tagged-human NGLY1 open reading frame driven by a CMV promoter (OriGene Technologies, Rockville, MD) was transduced into cells for the overexpression of NGLY1. A pLenti-C-Myc-DDK empty vector was used as the transduction control.

### Immunohistochemistry (IHC) and Fluorescence Staining

The general procedure for antibody-mediated fluorescence staining was previously described^12^ and provided as part of Supplementary Materials and Methods. The detailed information of primary antibodies was summarised in Supplementary Table S2.

### Immunoblotting

The general procedure for immunoblotting was described in a previously published report,^13^ except that cell lysates were prepared using M-PER mammalian protein extraction reagent (Thermo Fisher Scientific, Carlsbad, CA) containing EDTA-free protease inhibitor and phosphatase inhibitor cocktails (Millipore Sigma, St. Louis, MO). The detailed information of primary antibodies was summarised in Supplementary Table S2. HRP-conjugated secondary antibodies were from Jackson ImmunoResearch Laboratories (West Grove, PA).

### Flow Cytometry

The procedures were provided as part of Supplementary Materials and Methods.

### Cell Viability Test

The procedures were provided as part of Supplementary Materials and Methods.

### Gene Expression Analysis by qRT-PCR and Microarrays

The procedures for microarray analysis were provided as part of Supplementary Materials and Methods. The test of cellular pluripotency based on the transcriptomic features of cell samples was performed using the PluriTest (http://pluritest.org/).^14^ Multiplex qRT-PCR was performed using cDNA generated from the RNA samples and Taqman^®^ assays for the *NGLY1, FABP7*, *RSAD2*, *CCL5, IFNB1* and *ACTB* (internal control) genes (assay ID# Hs01046153_m1, Hs00361424_g1, Hs00369813_m1, Hs00982282_m1, Hs01077958_s1 and Hs03023943_g1; Thermo Fisher Scientific, Carlsbad, CA), according to the manufacturer’s instructions.

### Cytokine Profiling and Neutralisation

U-PLEX Human Interferon Combo assay kits and a SECTOR Imager 2400 (Meso Scale Discovery, Rockville, MD) were used to measure cytokine contents in conditioned medium samples of cells with indicated treatment, according to the manufacturer’s instructions. Specific antibodies against human INFβ1 and IL-29 (R&D Systems, Minneapolis, MN; Supplementary Table S2) were used to neutralise the cytokines in cell samples, while the IgG isotype (Jackson ImmunoResearch Laboratories, West Grove, PA) was applied to control samples.

### In vivo Studies

The animal work in this study was completed using an animal study service provided by the translational core laboratory at the University of Maryland, Baltimore. All experimental procedures and protocols utilizing mice were approved by the Institutional Animal Care and Use Committee at the University of Maryland. The procedures were provided as part of Supplementary Materials and Methods.

### Proteomics Analysis

The detailed procedures were provided as part of Supplementary Materials and Methods.

### Chemical Synthesis and Characterisation of NGLY1 Inhibitors

The detailed procedures of chemical synthesis and characterisation for the novel analogs of *N*-acetylglucosamine (GlcNAc)-linked asparagine were provided as part of Supplementary Materials and Methods. Z-VAD-fmk were purchased from Millipore Sigma (St. Louis, MO). WRR139 was synthesised and characterised according to the chemical approaches previously described.^15^

### Computational Modeling

The procedures were provided as part of Supplementary Materials and Methods.

### Production of recombinant human NGLY1 and RNase B deglycosylation assay

The procedures for generating recombinant human NGLY1 and testing its enzymatic activity were provided as part of Supplementary Materials and Methods.

### Statistical Analysis

The significance of differences in comparisons was primarily determined by the two-tailed Student’s *t*-test for a two-group comparison, unless stated otherwise in the figure legends. The association of NGLY1 staining results and pathological conditions in normal skin and melanoma tumour tissues was examined using a 2 × 2 contingency with the two-tailed Fisher’s exact test.

### Data and materials availability

The gene expression array data have been deposited with a link to an accession number GSE106936 in the Gene Expression Omnibus (GEO). Other data included within the article to support the findings of this study are available from the corresponding author upon reasonable request. The biological samples and novel compounds used in this study may be distributed upon request and under institutional material transferring agreements or a licensing process.

## Results

### NGLY1 is highly upregulated in melanoma cells but dispensable for the vitality and pluripotency of pluripotent stem cells

**Fig. 1:**
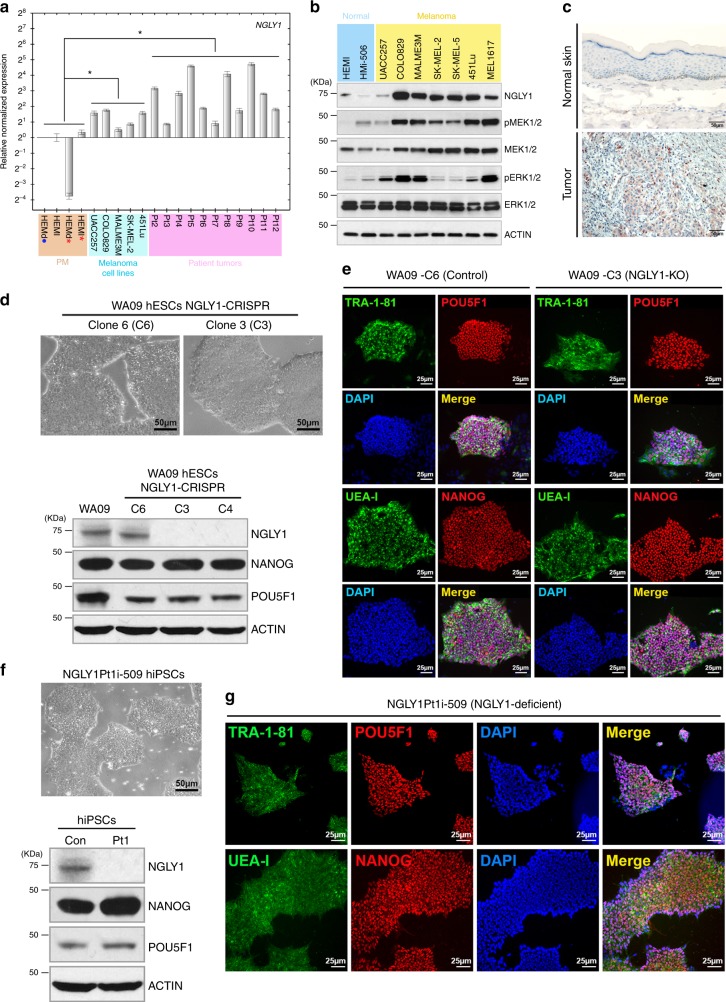
NGLY1 expression in normal and melanoma cells. (**a**) The expression of the *NGLY1* gene in cells at the transcriptional level was measured using qRT-PCR. *Orange shading:* primary melanocytes (PM). *Blue shading:* human melanoma cell lines. *Pink shading:* tumour samples of melanoma patients. *Blue dot:* undetectable NGLY1 transcript in the sample. *Red asterisk:* cell samples collected at high passage numbers ( > 16). All data were presented as mean ± standard deviation (*n* = 3; *P < 0.05, Mann–Whitney *U* test). The expression level of the *ACTB* gene in each sample was used as internal control for normalization. Gene expression levels in HEMl cells were used as comparison standards to calculate relative expression values. (**b**) The protein levels of NGLY1 detected using western blotting in cell samples. *Blue shading:* human normal cells. *Yellow shading:* human melanoma cell lines. (**c**) Representative images for immunohistochemistry staining of NGLY1 in normal skin and melanoma tumour tissues. With the same staining condition, the expression of NGLY1 was positively stained in the melanoma tumour tissue but not detected in the normal skin tissue. (**d**) WA09 hESC clones with and without the gene editing-mediated ablation of NGLY1 expression. *Upper panel:* cell morphology*. Lower panel:* the expression NGLY1 and pluripotency markers NANOG and POU5F1 detected by western blotting in the cells. *WA09:* parental WA09 hESCs. *WA09-C6:* a cell clone of WA09 hESCs derived from a gene-editing and selection process without acquiring disruptive mutations in the *NGLY1* gene. *WA09-C3 and WA09-C4:* two NGLY1-deficent cell clones of WA09 hESCs independently derived from a gene-editing and selection process. (**e**) The positive staining of pluripotency markers in WA09-C6 and WA09-C3 hESCs. (**f**) NGLY1Pt1i-509 hiPSCs established from cell reprogramming in NGLY1-deficient patient-derived dermal fibroblasts. *Upper panel:* cell morphology. *Lower panel*: the expression NGLY1 and pluripotency markers NANOG and POU5F1 detected by western blotting in the cells. (**g**) The positive staining of pluripotency markers in NGLY1Pt1i-509 hiPSCs

In contrast to human normal melanocytes, a majority of tested melanoma cell lines showed upregulation of NGLY1 (Fig. 1a, b). The significant upregulation of NGLY1 was also observed in patients’ tumour samples (Fig. 1a, c; Supplementary Table S3). Human PSCs, including undifferentiated hESCs and induced pluripotent stem cells (hiPSCs), are considered as normal cells in a unique cellular state, while similar to cancer cells regarding certain features.^16^ Normal melanocytes (HEMl)-derived hiPSCs, compared with HEMl cells, showed a similar expression level of NGLY1, much lower than that observed in melanoma cells (Fig. 1b). These findings suggest a potentially critical function of NGLY1 for cancer cells to sustain their viability or oncogenic signalling. Through gene editing, we obtained NGLY1-knockout (NGLY1-KO) clones and an editing-escaping (control) clone of WA09 hESCs. Despite the deficiency of NGLY1 observed in WA09-C3 and WA09-C4 hESCs (Fig. 1d), their morphology, viability and pluripotency were highly comparable to that of WA09-C6 and parental WA09 hESCs with NGLY1 expression (Fig. 1d, e; Supplementary Figure S1A and S1B). We also reprogrammed the NGLY1-deficient patient’s dermal fibroblasts into hiPSCs (Fig. 1f). Like the NGLY1-KO hESCs, the patient-derived hiPSCs without NGLY1 expression can be continuously cultured and maintain typical hPSC morphology, molecular features and the capacity of forming embryoid bodies (EBs) containing differentiated cells that are associated with three germ layers (Fig. 1g; Supplementary Figure S1C). Our results reveal that, while highly upregulated in melanoma cells, NGLY1 appears to be dispensable for the vitality of human normal cells even in a highly sensitive state like the embryonic stage.

### NGLY1 suppression disturbs proteasome-mediated protein degradation and induces stress response signalling-associated apoptosis in melanoma cells

**Fig. 2:**
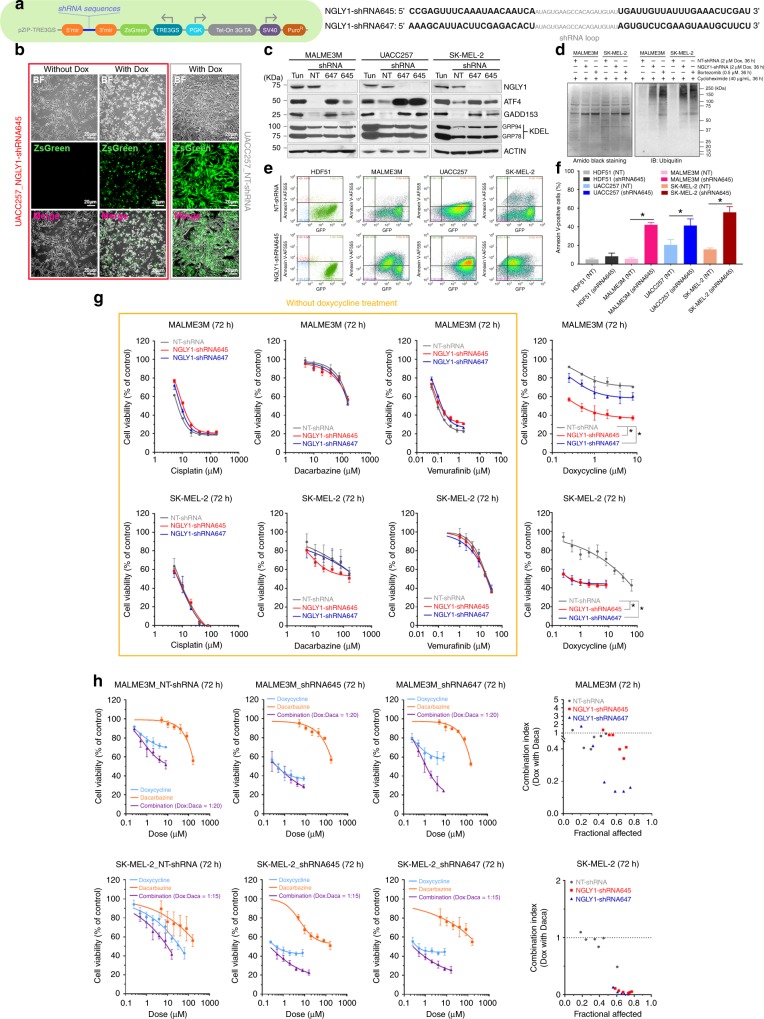
ER stress-associated apoptosis and synergistic anticancer responses induced by NGLY1 knockdown in melanoma cells. (**a**) The doxycycline (dox)-inducible pZIP-TRE3GS expression vector of non-targeting shRNA and NGLY1-targeting shRNA sequences. (**b**) The stable clones of UACC257 cells with dox-inducible shRNA. Cells with induced NGLY1-shRNA645 (green cells) showed morphological features of apoptosis, including shrinkage and fragmentation. Cells with induced non-targeting (NT)-shRNA maintain a morphology similar to the cells before dox induction. Cells were imaged after the treatment of 2 µM dox for 72 h. *BF:* bright field. *ZsGreen:* green fluorescence protein. (**c**) ATF4 and GADD153 signalling was activated by the shRNA-mediated knockdown of NGLY1 in melanoma cells. *Tun:* 2 µM tunicamycin for 24 h. (**D**) The accumulation of ubiquitinated proteins was detected using western blotting in MALME3M and SK-MEL-2 cells with NGLY1 knockdown. (**e**) The representative quadrant plots of flow cytometry analysis to detect apoptosis in normal (HDF51) and melanoma cells with NGLY1 knockdown. The cell samples were collected for analysis after the 72-hour induction of shRNA expression. (**f**) The quantitative results of flow cytometry analysis to detect apoptosis. All data were presented as mean ± standard deviation (*n* = 3; *P < 0.05, *t*-test) in the bar graph. (**g**) The dose-dependent suppression of viability in MALME3M and SK-MEL-2 cells with the indicated dox-inducible shRNA in response to cisplatin, dacarbazine, vemurafenib and dox treatment. All data were presented as mean ± standard deviation (*n* = 3, *P < 0.05, logistic regression). (**h**) The synergistic anticancer responses of NGLY1 knockdown and dacarbazine treatment for 72 h in MALME3M and SK-MEL-2 cells. The cell viability curves of combinatorial treatment were plotted according to the doses of dox used in the treatment. Combination indexes were calculated using Calcusyn software. A combination index value < 1 was considered synergistic. A combination index value < 0.2 was considered highly synergistic. All cell viability data were presented as mean ± standard deviation (*n* = 3)

Two independent NGLY1-targeting shRNA sequences (NGLY1-shRNA645 and NGLY1-shRNA647) and a non-targeting shRNA (NT-shRNA) sequence were cloned into doxycycline (dox)-inducible, polycistronic green fluorescence protein (GFP)-shRNA expression constructs (TransOMIC Technologies, Huntsville, AL; Fig. 2a). We generated the stable clones with the shRNA expression constructs in MALME3M, UACC257, SK-MEL-2, and COLO829 melanoma cells. The stable clones of melanoma cells showed clear GFP expression upon dox treatment (Fig. 2b), indicating the expression of shRNA sequences. Compared with the cells expressing the NT-shRNA, the expression of NGLY1 was largely suppressed by NGLY1-shRNA645 and NGLY1-shRNA647 and hardly detectable in the stable clones of MALME3M, UACC257 and SK-MEL-2 cells with 48-hour treatment of dox (Fig. 2c). Upon NGLY1 knockdown, melanoma cells showed morphological features of apoptosis, including shrinkage, fragmentation and detachment (Fig. 2b).

As shown in Fig. 2c, the NGLY1 knockdown-induced upregulation of ATF4 and GADD153 was detected in melanoma cells. In addition, NGLY1 knockdown hindered proteasome-mediated protein degradation, indicated by the accumulation of ubiquitinated proteins in cycloheximide-treated MALME3M and SK-MEL-2 cells (Fig. 2d). This finding is consistent with the previously observed suppression of ERAD in cells with NGLY1 malfunction.^1,4,5,15,17–19^ Since GADD153 is an important mediator for ER stress-associated apoptosis,^13^ our findings suggest that ER stress signalling-mediated apoptosis may contribute to the death of melanoma cells with NGLY1 suppression. Using flow cytometry analysis, a substantial increase of apoptosis was detected in NGLY1-knockdown melanoma cells, which was absent in the cancer cells expressing NT-shRNA and normal cells expressing NGLY1-targeting shRNA (Fig. 2e, f). Overexpression of exogenous human NGLY1 and knockdown of GADD153 both attenuated apoptosis induced by NGLY1 knockdown in SK-MEL-2 and UACC257 cells (Supplementary Figure S2). Taken together, stress response-associated, GADD153-mediated apoptosis contributes to NGLY1 knockdown-induced melanoma cell death.

### NGLY1 suppression sensitises melanoma cells to the treatment of DNA alkylating agents

Unlike the treatment of an ER stress inducer, tunicamycin, NGLY1 knockdown activates the transcription factor ATF4 and its downstream apoptotic factor GADD153 without upregulating ER chaperones GRP78/94 (Fig. 2c). Many chemotherapeutic drugs including DNA alkylating agents also induce GADD153 in cancer cells.^20^ Thus, NGLY1 suppression may synergise with DNA alkylating agents like dacarbazine and temozolomide to eliminate melanoma cells, at least partially, through intensified activation of GADD153. We tested whether NGLY1 suppression enhances the anticancer activity of dacarbazine and temozolomide that are commonly used to treat melanoma. Unlike MALME3M cells with the BRAF^V600E^ mutation and high sensitivity to vemurafenib,^21^ SK-MEL-2 cells with the NRAS^Q61R^ mutation^21^ are resistant to vemurafenib (Fig. 2g). The knockdown of NGLY1 compromised the viability of MALME3M and SK-MEL-2 cells (Fig. 2g) in viability assays where we also observed a highly synergistic effect of NGLY1 knockdown in combination with the cytotoxicity of either dacarbazine or temozolomide (Fig. 2h; Supplementary Figure S3 and S4). These results indicate that the suppression of NGLY1 could overcome melanoma cells with resistance to BRAF inhibitors as well as sensitise the cells to conventional chemotherapy agents that frequently lead to unsatisfactory outcomes in the treatment of patients with melanoma.

### Proteomic analysis reveals unique peptide signatures in melanoma cells in response to NGLY1 suppression

Upon NGLY1 suppression, ENGase has better access to the substrates and generates more products of glycopeptides containing GlcNAc-asparagine residues in cells (Supplementary Figure S5A).^1^ Using LC-MS/MS-based proteomics analysis, we have identified peptides containing GlcNAc-asparagine residues denoted as N(HexNAc) in both control and NGLY1-knockdown samples (Supplementary Figure S5B and S5C). Although the quantities of peptides containing GlcNAc-asparagine that can be detected among the biological replicates of different cell samples appeared to vary, compared with control cells, melanoma cells with NGLY1 knockdown reproducibly showed higher contents of peptides containing GlcNAc-asparagine residues, indicating the functional defect of NGLY1 in the cells (Supplementary Figure S5D). Many proteins also presented differential abundance in melanoma cells in response to NGLY1 knockdown (Supplementary Table S4). Among the proteins with reduced abundance in the NGLY1-knockdown cells, several of them (*e.g*., VCP, PDIA4, HSPA5 and HIST1H4A) have been linked to the survival and drug resistance of cancer cells.^22–25^ Thus, part of the anti-melanoma responses associated with NGLY1 inhibition may be attributed to the modulation of these gene products.

### Global gene expression profiling uncovers pleiotropic effects of NGLY1 suppression on melanoma cells

**Fig. 3:**
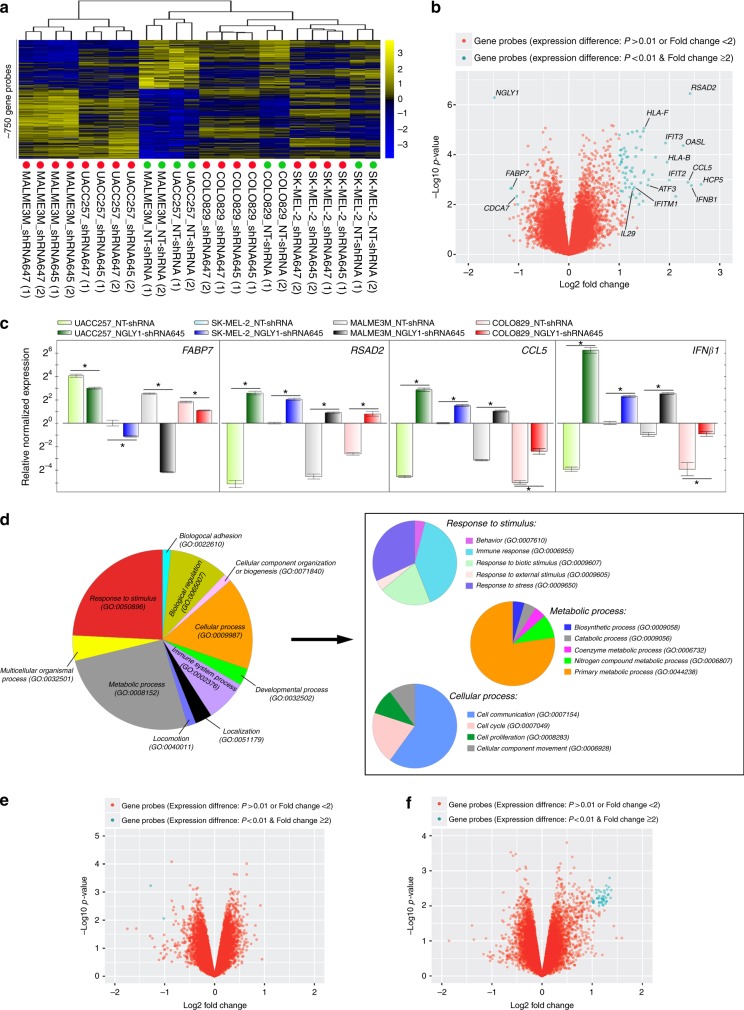
Differential gene expression caused by NGLY1 suppression in melanoma cells, hESCs and the differentiated derivatives of hESCs. SK-MEL-2, COLO829, UACC257 and MALME3M melanoma cells with the expression of the indicated inducible shRNA due to the treatment of 2 µM dox for 48 h were collected for RNA isolation and global gene expression profiling. WA09, WA09-C6, WA09-C3, WA09-C4 hESCs and their differentiated derivatives were also collected for analysis. Samples of two biological replicates for each setting were analysed. (**a**) A heat map representation of ~750 probes that measured the relative expression levels of differentially expressed genes (*P* < 0.01, *t*-test between control and knockdown cells) in melanoma cell samples expressing the indicated shRNA. *Red dots:* melanoma cells with NGLY1 knockdown. *Green dots:* control cells. (**b**) Selected genes that were differentially expressed (*P* < 0.01 and fold change ≥ 2) in the control and NGLY1-knockdown melanoma cells were annotated in a volcano plot of fold change vs. significance. (**c**) The qRT-PCR validation of selected genes that were differentially expressed in the control and NGLY1-knockdown melanoma cells (*n* = 3, *P < 0.05, *t*-test). The expression level of the *ACTB* gene in each sample was used as internal control for normalisation. Gene expression levels in SK-MEL-2 cells with NT-shRNA were used as comparison standards to calculate relative expression values. (**d**) Gene ontology analysis revealed that genes differentially expressed (*P* < 0.01 and fold change ≥ 2) due to NGLY1 suppression in melanoma cells were highly enriched in multiple biological processes. (**e**) A volcano plot of fold change vs. significance for selected genes that were differentially expressed (*P* < 0.01 and fold change ≥ 2) in control and NGLY1-deficient WA09 hESCs. (**f**) A volcano plot of fold change vs. significance for selected genes that were differentially expressed (*P* < 0.01 and fold change ≥ 2) in the embryoid bodies of control and NGLY1-deficient WA09 hESCs gone through 6 days of non-directed differentiation

Using transcriptomic analysis, we identified a group of genes (~750 gene probes corresponding to ~700 genes) that were significantly (*P* < 0.01) and commonly upregulated or downregulated between control and NGLY1-knockdown melanoma cells. The hierarchical clustering of all the cell samples based on the expression of these genes showed that, within the same cell line, all the NGLY1-kockdown samples were similar and segregated from the control samples (Fig. 3a). Data analysis with an additional filtering criterion (expression fold change ≥ 2) showed that NGLY1 suppression appeared to primarily induce gene upregulation. Many of these upregulated genes, including the *IFNβ1* and *IL-29* genes, are highly associated with cytokine responses in cells (Fig. 3b). The expression of differentially expressed genes was also validated using qRT-PCR (Fig. 3c). In addition to the cytokine signalling-relevant genes, many genes like *XAF, ATF3, PMAIP1* (*NOXA*), *AXUD1* and *CDKN2C* that have been liked to anticancer activity^26–31^ were significantly upregulated in the NGLY1-knockdown cells, while genes like *FABP7*, *CRYAB* and *GAPDHS* that have been associated with the survival, proliferation and invasiveness of cancer cells or with a poor prognosis in melanoma patients^32–34^ were significantly downregulated (Supplementary Table S5). Ontology analysis showed that ~60 differentially expressed genes (*P* < 0.01, expression fold change ≥ 2) are highly involved in multiple biological processes (Fig. 3d), including immune and stress responses, primary metabolic process, cell communication, and cell cycle. In contrast to substantial perturbation induced by NGLY1 knockdown in melanoma transcriptomes, the disruption of NGLY1 expression in normal hPSCs and their differentiated derivatives caused limited changes in their gene expression networks (Fig. 3e, f). None of the differentially expressed genes (*P* < 0.01, expression fold change ≥ 2) identified in NGLY1-knockdown melanoma cells showed significant expression alterations in NGLY1-deficient hPSCs and their differentiated derivatives, highlighting the fundamental differences of normal and malignant cells in response to NGLY1 inhibition.

### NGLY1 suppression induces cytokine surges that contribute to melanoma cell death

**Fig. 4:**
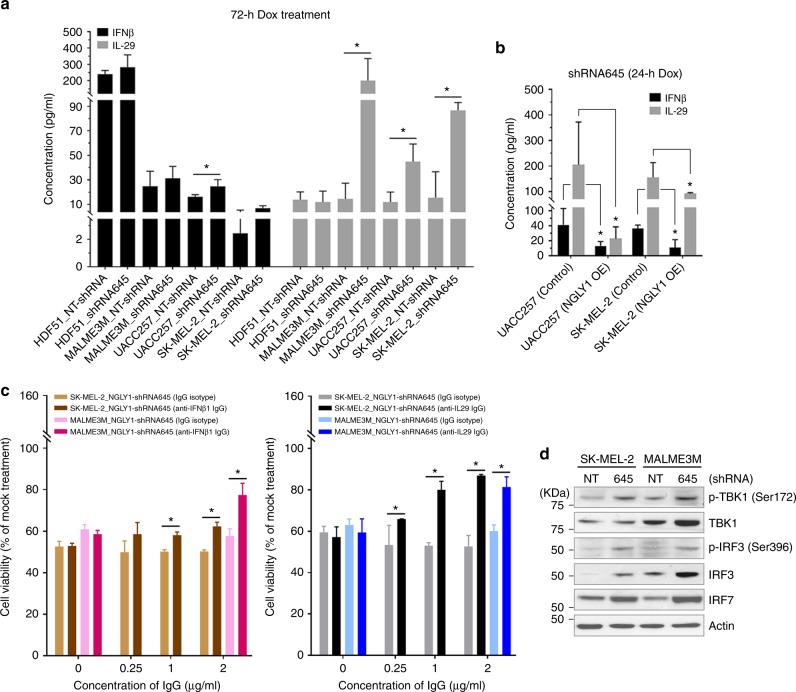
NGLY1 suppression enhanced the production of IFNβ1 and IL-29 that contributes to viability reduction in melanoma cells. (**a**) The contents of IFNβ1 and IL-29 in the conditioned media of UACC257 and SK-MEL-2 cell clones with the indicated treatment were measured by cytokine profiling. (**b**) The NGLY1 knockdown-induced upregulation of IFNβ1 and IL-29 was significantly attenuated by the overexpression of exogenous human NGLY1 in the cells. (**c**) *Left panel:* the attenuation of NGLY1 knockdown-induced viability reduction by the treatment of specific IFNβ1 neutralisation antibody in the cells. *Right panel:* the attenuation of NGLY1 knockdown-induced viability reduction by the treatment of specific IL-29 neutralisation antibody in the cells. NGLY knockdown was induced by the treatment of 2 µM dox for 72 h in the cells. (**d**) The enhanced expression and activation of IRF3, IRF7 and their upstream kinase TBK1 was detected in SK-MEL-2 and MALME3M cells with NGLY1 knockdown. The serine phosphorylation of IRF3 and TBK1 indicates their activity. *NT:* non-targeting shRNA. *645:* NGLY1-targeting shRNA645. All data were presented as mean ± standard deviation (*n* = 3; *P < 0.05, *t*-test)

In contrast to the conditioned medium of control cells, the conditioned medium of NGLY1-knockdown melanoma cells contained a significantly higher amount of IFNβ and/or IL-29 (Fig. 4a), consistent with our findings from gene expression profiling. The overexpression of exogenous human NGLY1 significantly attenuated the NGLY1 knockdown-mediated induction of IFNβ and IL-29 in melanoma cells (Fig. 4b). Using IFNβ1- and IL-29- neutralisation antibodies but not their IgG isotypes, we partially rescued the NGLY1 knockdown-triggered inhibition of viability in SK-MEL-2 and MALME3M cells (Fig. 4c). In addition, a considerable overlap was found between the differentially expressed genes in melanoma cells with IL-29 treatment^35^ and NGLY1 knockdown (Supplementary Table S5). Notably, the anti-melanoma activity of IFNβ and IL-29 has been demonstrated in previous studies.^35–37^ These findings definitively illustrate that NGLY1 suppression-induced cytokine surges and relevant cellular responses contribute to anticancer effects. Unlike melanoma cells, human normal fibroblasts showed no significant changes in the production and release of IFNβ and IL-29 in response to NGLY1 knockdown (Fig. 4a).

### NGLY1 suppression upregulates transcription factors that activate the expression of interferon λ genes in melanoma cells

By mapping ~1400 manually curated, sequence-specific DNA-binding transcription factors^38^ to our global gene expression profiling data, we discovered that the expression of 155 transcriptional factor genes was altered (*P* < 0.05) due to NGLY1 knockdown in melanoma cells. Among these differentially expressed transcription factors, we found that the average RNA expression levels of IRF1, IRF3, IRF7, IRF9, REL (an NF-κB subunit) and RELB were elevated in the NGLY1-knockdown cells by 2.41, 1.25, 2.23, 1.66, 1.12 and 1.65 folds, respectively. In addition, the expression and activation^39,40^ of IRF3 and IRF7 proteins was enhanced in melanoma cells with NGLY1 knockdown (Fig. 4d). Since the expression of the interferon λ gene family members including the *IL-29* (interferon λ1) gene is primarily regulated by the IRF proteins and NF-κB,^41^ it is likely that NGLY1 suppression causes robust upregulation of IRF and NF-κB transcription factors and subsequently upregulates IL-29 expression in melanoma cells.

### NGLY1 suppression hinders melanoma tumour growth in vivo

**Fig. 5:**
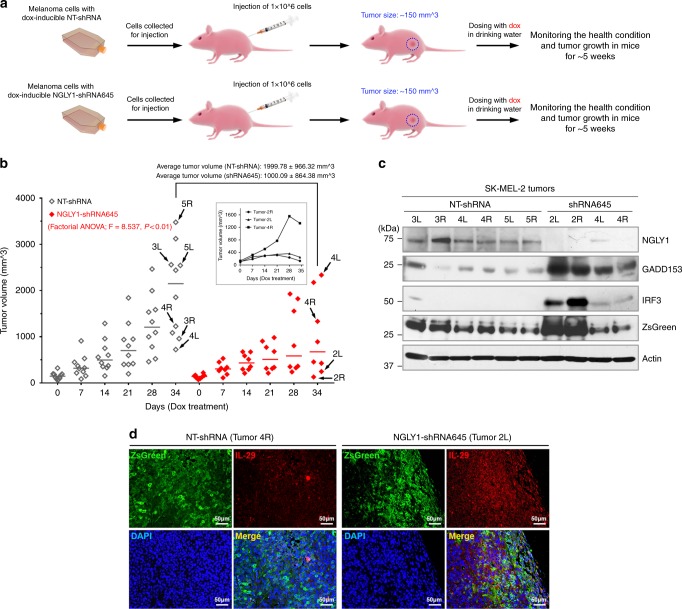
The in vivo antitumour activity of targeting NGLY1 in melanoma cells. (**a**) A schematic illustration of animal study design to test the in vivo antitumour efficacy of NGLY1 suppression in melanoma. (**b**) The volume changes of xenografted SK-MEL-2 tumours with the induction of NT-shRNA (*n* = 10) and NGLY1-shRNA645 (*n* = 8) for 35 days. NGLY1 knockdown was a significant factor that affected the tumour volume (Factorial ANOVA; *F* = 8.537, *P* < 0.01). Tumours were harvested at the end of the study for western blotting analysis. *Bars:* median tumour volumes at the indicated time points. *Inset:* the volume changes of three tumours with NGLY1-targeting shRNA that initially increased their size but showed regression at the end of the study. (**c**) The expression of NGLY1, GADD153, IRF3, and GFP (ZsGreen) proteins in selected tumours was analysed by western blotting. (**d**) The enhanced expression of IL-29 in the tumour tissues with NGLY1 knockdown was detected by immunofluorescence staining

Using a xenograft tumour model (Fig. 5a), we test the anti-melanoma response of NGLY1 suppression. The growth of melanoma tumours established with SK-MEL-2 cells in mice was impeded by the induced knockdown of NGLY1 (Fig. 5b, c). It was also noticed that three out of eight tumours with NGLY1-targeting shRNA that increased their size during the initial 3-4 weeks of dox treatment showed regression at the end of the study (Fig. 5b, **inset**). The enhanced expression of GADD153, IRF3 and IL-29 was also detected in the NGLY1-knockdown tumours (Fig. 5c, d). These findings attest to the in vivo antitumour efficacy of NGLY1 inhibition that was expected from our in vitro studies.

### Novel NGLY1 inhibitors show an anti-melanoma efficacy with a limited impact on normal cells

**Fig. 6:**
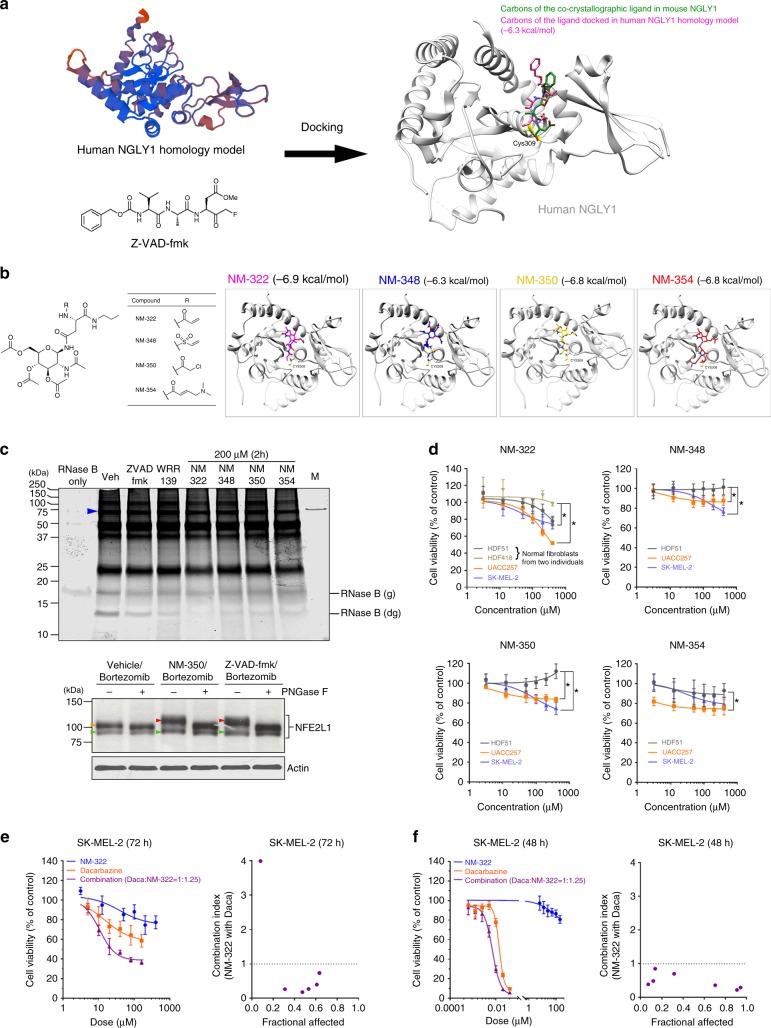
Anticancer responses induced by novel covalent modifiers that target the catalytic site of human NGLY1 in melanoma cells. The computational homology model of human NGLY1 core domain was generated and used for studying interactions between NGLY1 and novel small molecules that are designed to covalently modified and inactivate the catalytic site of NGLY1. (**a**) The most favourable binding pose of Z-VAD-fmk, a short peptide with NGLY1 and caspase inhibitory activity, in the human NGLY1 homology model superimposed to the conformation of Z-VAD-fmk bound to mouse NGLY1 in a co-crystalised structure. (**b**) Novel small molecules (NM-322, NM-348, NM-350, and NM-354) that mimic a GlcNAc-conjugated asparagine in the NGLY1 substrates of NGLY1 and contain strategically positioned electrophilic groups bound to the human NGLY1 homology model in computational docking and showed their high binding affinities with the electrophilic groups pointed towards Cys309 in close proximity at the human NGLY1 catalytic site. (**c**) *Upper panel:* the 2-hour reaction of covalent modifiers, including Z-VAD-fmk (20 µM), WRR139 (5 µM), NM-322, NM-348, NM-350 and NM-354, with human NGLY1 suppressed its activity in the deglycosylation of denatured RNase B. *Blue arrowhead:* recombinant NGLY1-FLAG. *RNase B (g):* glycosylated RNase B. *RNase B (dg):* deglycosylated RNase B. *Veh:* vehicle (DMSO) treatment. *M:* molecular weight marker. *Lower panel:* the deglycosylation of NFE2L1 altered by the treatment of 20 µM Z-VAD-fmk and 200 µM NM-350 in bortezomib-treated HEK293T cells. The cells were pretreated with vehicle (DMSO), Z-VAD-fmk and NM-350 for 24 h and subsequently subjected to concomitant treatment with 10 µM bortezomib for an additional 16 h. Cell lysates reacted with and without 500 units of PNGase F for 2 h were analysed using western blotting. *Red arrowhead:* fully glycosylated NFE2L1. *Orange arrowhead:* partially glycosylated NFE2L1. *Green arrowhead:* deglycosylated and truncated NFE2L1. (**d**) The dose-dependent suppression of cell viability was preferentially induced by the novel NGLY1 inhibitors in melanoma cells compared with normal cells (*P < 0.05, logistic regression). (**e**) The synergistic effect was observed between NM-322 and dacarbazine in the suppression of melanoma cell viability. The cell viability curve of combinatorial treatment was plotted according to the doses of dacarbazine used in the treatment. (**f**) The synergistic effect was observed between NM-350 and bortezomib in the suppression of melanoma cell viability. The cell viability curve of combinatorial treatment was plotted according to the doses of bortezomib used in the treatment. All the data of cell viability tests were presented as mean ± standard deviation (*n* = 3)

Due to the lack of optimised small molecules that specifically inactivate human NGLY1, we aimed to develop novel NGLY1 inhibitors and test their potential anti-melanoma use. Based on the crystal structure of mouse NGLY1 (PDB code: 2F4M)^42^ as a model-building template, we built a homology model of the human NGLY1 core domain (Fig. 6a) using a homology-modelling web server SWISS-MODEL. To test our human NGLY1 homology model, we used it to perform *in silico* docking analysis of known molecules that may inactivate human NGLY1. Since Z-VAD-fmk (a benzyloxycarbonyl-Val-Ala-Asp tripeptide with fluoromethyl group at the C-terminal) penetrates cells and reacts with yeast, mouse and human NGLY1^15,42,43^ by forming a covalent bond with the cysteine residues at the catalytic sites (*e.g*., Cys309 in human NGLY1), we docked Z-VAD-fmk to the human NGLY1 homology model using AutoDock. As expected, the top-scored binding poses of Z-VAD-fmk in our homology model include the ones that are similar to the binding pose in the crystal structure of a mouse NGLY1 and Z-VAD-fmk complex (PDB code: 2F4O; Fig. 6b), indicating that Z-VAD-fmk can bind to and inactivate the catalytic site of human NGLY1 in a similar fashion. This result also supports the accuracy and suitability of our NGLY1 homology model for inhibitor screening.

We designed four small molecules (NM-322, NM-348, NM-350 and NM-354) that mimicked the *N*-acetylglucosamine (GlcNAc)-linked asparagine substrates of NGLY1 and contained strategically positioned electrophilic groups (Fig. 6b). Their preferred binding poses in the human NGLY1 homology model included catalytic poses where the electrophilic reactive moieties of compounds were oriented toward Cys309. The binding energy of these compounds in their most favourable catalytic poses ranged from -6.3 to -6.9 kcal/mol. Since the binding energy of the most favourable binding pose of Z-VAD-fmk in the homology model was estimated as -6.3 kcal/mol, considering the ± 2.0 kcal/mol variation of binding energy calculation in AutoDock, it is believable that our novel compounds could have comparable inhibitory activity towards human NGLY1 as Z-VAD-fmk. Like Z-VAD-fmk and WRR139,^15^ our compounds caused NGLY1 inhibition and blocked the deglycosylation of denatured RNase B in vitro (Fig. 6c, **upper panel**). Since NGLY1 inactivation hinders the deglycosylation and proteolytic processing of NFE2L1^15^ in proteasome inhibitor-treated cells, we tested whether our novel inhibitors may interfere with NFE2L1 deglycosylation. We used HEK293T cells (a sub-clone of HEK293 cells) in this test because HEK293 cells have been used in a similar study and appear to tolerate NGLY1 suppression well.^15^ HEK293T cells pretreated with NM-350 showed clear retention of *N-*glycans on NFE2L1, indicated by the electrophoretic mobility shift of full-length NFE2L1 (Fig. 6c, **lower panel**). The electrophoretic mobility shift of NFE2L1 due to glycan retention was also detected in NGLY1-deficient hPSCs treated with bortezomib (Supplementary Figure S1D).

As shown in Fig. 6d, our novel compounds preferentially inhibited melanoma cell viability and had limited impact on normal cells. The treatment of our inhibitors also enhanced the production and release of IFNβ and IL-29 in melanoma cells but not normal cells (Supplementary Figure S6A). Similar to the synergism between NGLY1 knockdown and dacarbazine treatment, NM-322 and dacarbazine caused a synergistic effect on the suppression of SK-MEL-2 cells (Fig. 6e). Consistent with the potentiation of proteasome inhibitor cytotoxicity caused by WRR139-mediated NGLY1 inhibition,^15^ NM-350 and bortezomib synergistically suppressed the viability of SK-MEL-2 cells (Fig. 6f). These results highlight that pharmacological inactivation of NGLY1 reduces melanoma cell viability and may be exploited for cancer therapy purposes.

### Melanoma cells present similar peptide signatures in response to NGLY1 knockdown and novel NGLY1 inhibitors

Having elevated contents of GlcNAc-asparagine-containing peptides and the differential abundance of specific proteins detected in NGLY1-knockdown melanoma cells (Supplementary Figure S5; Supplementary Table S4), we examined proteomic changes in melanoma cells treated with our NGLY1 inhibitors. Like the NGLY1-knockdown cells, cells treated with our inhibitors showed relatively high contents of GlcNAc-asparagine-containing peptides (Supplementary Figure S6B), suggesting that our inhibitors are likely to suppress NGLY1 activity and allow ENGase to generate more enzymatic products with the GlcNAc-asparagine signature. Similar alteration patterns associated with the differentially abundant proteins identified in the NGLY1-knockdown cells were observed in melanoma cells treated with NM-350 (Supplementary Table S4), further supporting the NGLY1-inhibitory and anticancer activity of our novel compounds.

## Discussion

In this study, we revealed the significance and molecular mechanisms of NGLY1 as a novel target in melanoma for the induction of cancer cell-specific apoptosis and the development of new anticancer approaches.

From studying a genetic disorder known as NGLY1 deficiency, we realised that critical organs maintain their necessary functions for the vitality of NGLY1-deficient individuals.^5,9^ Although many types of cells present abnormal features in patients with NGLY1-deficiency, these abnormalities may be largely attributed to abnormal embryonic development rather than the direct effects of NGLY1 loss on terminally differentiated somatic cells. Thus, the impact of NGLY1 suppression on differentiated somatic cells could be quite mild. We indeed observed that NGLY1 suppression leads to limited perturbation in human normal cells while causing detrimental outcomes in melanoma cells.

Through proteomic analysis, we not only observed a higher content of peptides containing GlcNAc-asparagine residues but also identified proteins showing altered abundance in melanoma cells with NGLY1 inhibition (Supplementary Table S4). However, in transcriptomic analysis, the genes that encode several of these differentially abundant proteins (*e.g*., PPIA, VDAC1, PRKCSH, LASP1) did not appear differentially expressed at the RNA level in the NGLY1-inhibited cells, indicating that NGLY1 inhibition may affect cell signalling networks by perturbing post-transcriptional or post-translational regulatory mechanisms. The links between NGLY1 and post-translational regulatory mechanisms were also supported by the findings from several recent studies on NGLY1.^15,17–19^

Although ATF4 and GADD153 were upregulated in NGLY1-knockdown melanoma cells, we did not observe a clear upregulation of ER chaperones GRP78/94 (Fig. 2c). In fact, the abundance of GRP78/94 proteins in melanoma cells determined by mass spectrometry appeared to drop in response to NGLY1 suppression (Supplementary Table S4). The missing of GRP78/94 upregulation suggests that the NGLY1 suppression-induced activation of ATF4 and GADD153 in melanoma cells may not be directly caused by ER stress. Since ER stress caused by the disruption of protein folding and ER function typically activates three arms of signalling pathways downstream of ER membrane-bound proteins that include PERK, ATF6, and IRE1α, we also examined the activation of these three signalling arms in NGLY1-knockdown melanoma cells. The enhanced phosphorylation of eIF2α was observed in melanoma cells due to the treatment of tunicamycin and NGLY1 knockdown (Supplementary Figure S2F), indicating the activation of the eIF2α signalling as the upstream of ATF4. The activation of both ATF6 and XBP1, however, was not seen in the NGLY1-knockdown cells (Supplementary Figure S2F). Interestingly, we detected the enhanced phosphorylation of PKR and PERK in the NGLY1-knockdown cells (Supplementary Figure S2G). These findings further support that the integrated stress response signalling,^44^ rather than ER stress, could be a key mediator for the NGLY1 suppression-induced activation of ATF4 and GADD153 in melanoma cells.

Through global gene expression profiling (Fig. 3), we discovered that, in addition to the activation of stress-response signalling, NGLY1 suppression in melanoma cells causes the significant upregulation of interferon genes that have well documented anticancer activity. Our data (Fig. 4) demonstrated that these cytokine surges play an important role in melanoma cell death as the consequence of NGLY1 inhibition. NGLY1 suppression-stimulated cytokine responses also offer direct evidence supporting the feasibility of immunomodulation, particularly in malignant cells, by targeting NGLY1. Thus, NGLY1 suppression may potentiate the anticancer activity of immune-modulatory agents. A recent study has revealed that the chemical-induced activation of type I interferon (*e.g*., Ifnb1) signalling and immunogenic cell death potentiates the antitumour efficacy of anti-PD-1 antibody in syngeneic mouse tumour models of colon and bladder cancer.^45^ Since anti-PD-1 antibodies such as nivolumab and pembrolizumab are used for treating patients with melanoma and non–small cell lung cancer, it is reasonable to consider the induction of cytokine surges by targeting NGLY1 as a promising approach to enhance the efficacy of immune checkpoint therapies in these patients. In the future, the immune responses triggered by NGLY1 inhibition may be exploited for antiviral purposes because the NGLY1 suppression-activated cytokine signalling is also relevant to antiviral responses in cells.^46^

We computationally modelled the conformations and interactions of human NGLY1 with different potential inhibitors. Like our newly designed inhibitors, WRR139 showed a decent binding affinity with its electrophilic reactive moiety oriented towards Cys309 at the catalytic domain of human NGLY1 (Supplementary Figure S7A). Although WRR139 and our novel compounds may both effectively bind to human NGLY1, their binding poses in NGLY1 were distinct (Supplementary Figure S7B). This difference suggests that there could be sufficient space at the catalytic site of NGLY1 to permit additional possibilities for compound derivation and optimisation to ultimately achieve a competitive inhibitor with high specificity and other desired features for clinical applications.

Our results support a conclusion that human normal and melanoma cells present distinct features in response to NGLY1 suppression. In addition, NGLY1 suppression generates multifaceted anticancer responses (Supplementary Figure S8) in vitro and in vivo. Our discoveries serve as a vivid proof for the high value of understanding the biology hinted at by a rare genetic disorder like NGLY1 deficiency. Reciprocally, our findings about NGLY1 suppression-caused cytokine responses in diseased cells indicate that immunity alterations may occur in the NGLY1-deficient patients whose development, health conditions, and susceptibility to disease or pathogens require close monitoring. We believe that continuous studies regarding NGLY1 function in normal development and pathological conditions are likely to shed light on new strategies for managing multiple human disorders.

## Electronic supplementary material

Supplementary Information

## Availability of data and material

Upon appropriate request with the completion of interinstitutional material transferring agreements/licensing, the cell clones and novel compounds used in this study could be made available for other researchers’ use. The data of global gene expression profiling have been deposited in an open access database. The detailed information was provided under **Materials and Methods**.
