# Supplementary material for: Stress and interferon signalling-mediated apoptosis contributes to pleiotropic anticancer responses induced by targeting NGLY1
Source: Br J Cancer. 2018 Nov 2;119(12):1538–51. doi: 10.1038/s41416-018-0265-9 (PMC6288164; doi:10.1038/s41416-018-0265-9)
Supplement: Supplementary file 1 — Supplementary Information [file 41416_2018_265_MOESM1_ESM.pdf]

Stress and interferon signaling-mediated apoptosis contributes to pleiotropic anticancer responses induced by targeting NGLY1

Ashwini Zolekar[1†], Victor J.T. Lin[1†], Nigam M. Mishra[1], Yin Ying Ho[2], Hamed S. Hayatshahi[1], Abhishek Parab[3], Rohit Sampat[1], Xiaoyan Liao[4], Peter Hoffmann[2,5], Jin Liu[1], Kyle A. Emmitte[1], Yu-Chieh Wang[1]*

[1] Department of Pharmaceutical Sciences, UNT System College of Pharmacy, University of North Texas Health Science Center, Fort Worth, TX, USA

[2] Adelaide Proteomics Centre, The University of Adelaide, Adelaide, Australia

[3] Department of Mathematics, Purdue University, West Lafayette, Indiana, USA

[4] Department of Pathology, University of California, San Diego, San Diego, California, USA

[5] Future Industries Institute, University of South Australia, Adelaide, Australia

*To whom correspondence should be addressed:

Yu-Chieh Wang, Ph.D.
Department of Pharmaceutical Sciences
The University of North Texas Health Science Center
3500 Camp Bowie Boulevard, RES-314G
Fort Worth, Texas 76107
Tel: 1-(817) 735-2944
Fax: 1-(817) 735-2603
Email: yu-chieh.wang@unthsc.edu

†A.Z. and V.J.T.L. equally contributed to this work.

Supplementary Information:

Materials and Methods

Knockdown of NGLY1 and GADD153 (DDIT3)

NGLY1-shRNA645: $^{5'}$CCGAGUUUCAAAUAACAAUCAAUAGUGAAGCCACAGAUGUAUUGAUUGUUAUUUGAAACUCGAU$^{3'}$, NGLY1-shRNA647: $^{5'}$AAAGCAUUACUUCGAGACACUAUAGUGAAGCCACAGAUGUAUAGUGUCUCGAAGUAAUGCUUCU$^{3'}$, DDIT3-shRNA301: $^{5'}$AAGGUCCUGUCUUCAGAUGAAAUAGUGAAGCCACAGAUGUAUUUCAUCUGAAGACAGGACCUCU$^{3'}$, DDIT3-shRNA303: $^{5'}$AGAGAAAGAACAGGAGAAUGAAUAGUGAAGCCACAGAUGUAUUCAUUCUCCUGUUCUUUCUCCU$^{3'}$, DDIT3-shRNA304: $^{5'}$AGUCCUGUCUUCAGAUGAAAAAUAGUGAAGCCACAGAUGUAUUUUUCAUCUGAAGACAGGACCU$^{3'}$

Immunohistochemistry (IHC) and Fluorescence Staining

For the staining of pluripotency biomarkers in hPSCs and their differentiated derivatives, cells were plated into 24-well plates, fixed and permeabilized and incubated with primary antibodies against specific pluripotency biomarkers and fluorophore-conjugated secondary antibodies (Thermo Fisher Scientific, Carlsbad, CA). For the IHC staining of FFPE tissue sections, tissue sections were dewaxed, rehydrated, subjected to antigen retrieval using a universal antigen retrieval reagent (R&D Systems, Minneapolis, MN), and reacted with a primary antibody against human NGLY1 (Millipore Sigma, St. Louis, MO) at 4°C for overnight. After thorough washing with PBS containing 0.2% Tween-20 (PBST; Millipore Sigma, St. Louis, MO), the tissue samples were reacted with a HRP-conjugated secondary antibody (Jackson ImmunoResearch Laboratories, West Grove, PA) at room temperature for 2 hours, washed with PBST, processed using an AEC peroxidase substrate kit (Vector Laboratories, Burlingame, CA) and subsequently stained with Mayer’s hematoxylin solution (Millipore Sigma, St. Louis, MO). The stained tissue samples were mounted with cover slips and read by a pathologist who is experienced in the identification of cancer cells in tissue sections with IHC staining.

Flow Cytometry

For quantifying the percentages of apoptotic cells in cell samples, samples (~1 x 10^6 cells per sample) stained with Annexin V-Alexa Fluor 647 (Thermo Fisher Scientific, Carlsbad, CA) according to the manufacturer's instruction were analyzed using a SH800Z cell sorter (Sony Biotechnology, San Jose, CA). In addition to the Annexin V-Alexa Fluor 647, anti-FLAG rat IgG-Alexa Fluor 555 (Thermo Fisher Scientific, Carlsbad, CA) was used for labeling fixed cells with the overexpression of FLAG-tagged human NGLY1 prior to cytometry analysis in the rescue study.

Cell Viability Test

Cells were seeded into 96-well plates (2,500-5,000 cells/well, depending on cell types), incubated overnight, and treated as indicated. If DMSO was used as a vehicle to dissolve compounds and generate stock compound solutions for drug treatment, control groups received DMSO (0.1%, final concentration). After treatment, cells were incubated in FBS-free medium containing 0.4 mg/mL MTT (3-[4,5-dimethyl- thiazol-2-yl]-2,5-diphenyl-2*H*-tetrazolium bromide; TCI America, Portland, OR) at 37°C for 1 hour. Reduced MTT was solubilized in DMSO for determination of absorbance at 570 nm. Absorbance of reduced MTS was directly measured in the reaction medium at 490 nm. The relative cell viability in each treatment condition was calculated based on absorbance values. The combination indices (C.I.) of cell viability suppression induced by combinatorial treatment were calculated using Calcusyn 2.0. C.I. values less than 1 are generally considered as synergistic effects from the combinatorial treatment. The lower a C.I. value gets, the stronger the synergistic effect is.

Gene Expression Analysis by qRT-PCR and Microarrays

Total RNA was isolated from cell samples using the mirVana miRNA Isolation Kit (Thermo Fisher Scientific, Carlsbad, CA). The quality of each RNA samples was determined using an Agilent 2200 Tape Station system (Agilent, Santa Clara, CA) for RNA integrity analysis. Samples with RIN^e numbers above 7 were chosen to move forward with global gene expression profiling. The iScript Reverse Transcription Supermix (Bio-Rad, Hercules, CA) was used to generate the cDNA of total RNA samples. Global gene expression profiling was performed using HT-12v4 Human Gene Expression Bead Chips and a HiScan array scanning system (Illumina, Hayward, CA), according to the manufacturer's instructions. The gene expression

array data have been deposited with links to an accession number GSE106936 in the Gene Expression Omnibus (GEO). Data were filtered for detection *P* value <0.01 in GenomeStudio (Illumina, Hayward, CA), and normalized using the LUMI package with RSN (Robust spline normalization) algorithm in R. The limma package in R was used for multivariate analysis to identify the top differentially expressed genes (*P*<0.01). The pheatmap package was used for clustering analysis and generating heat map representations in R. The volcano plots were obtained using the limma package in conjunction with the ggplot2 package in R. The ontology analysis of differentially expressed genes was performed using the PANTHER 12.0 (http://pantherdb.org/about.jsp).

In vivo Studies

Six-week-old female NOD.CB17-*Prkdc*^{scid}/J mice (The Jackson Laboratory, Bar Harbor, ME) were group-housed under conditions of constant photoperiod (12 hours light: 12 hours dark) with *ad libitum* access to sterilized food and water. Since the animal work in this study was completed using an animal study service provided by the translational core laboratory at the University of Maryland, Baltimore, all experimental procedures and protocols utilizing mice were approved by the Institutional Animal Care and Use Committee at the University of Maryland. The clones of SK-MEL-2 cells that carry inducible NT-shRNA and NGLY1-shRNA645 were used in the animal studies. Each mouse was subcutaneously inoculated with 1 $\times 10^6$ cancer cells in a total volume of 0.1 mL serum-free medium containing 50% Matrigel (Corning, Tewksbury, MA). As tumors became established (mean starting tumor volume: 154.2 ± 78.3 mm^3 for NT-shRNA and 141.8 ± 48.2 mm^3 for NGLY1-shRNA645) in mice, their *ad libitum* access to water was discontinued. Subsequently, sterilized water containing 0.5 mg/ml doxycycline and 5% sucrose freshly prepared every other day in bottles was provided to the tumor-bearing animals for 5 weeks. Mice bearing tumors (n=10 for NT-shRNA and n=8 for NGLY1-shRNA645) were included in the study. Tumors were measured weekly using calipers and their volumes calculated using a standard formula: width^2 x length x 0.52. Body weights were measured weekly. At terminal sacrifice, complete necropsies were performed on all mice and tumors were harvested. A portion of each tumor was frozen in liquid nitrogen for western blotting analysis and the remainder was fixed in 10% formalin for immunohistochemical or immunofluorescence staining purposes.

Production of recombinant human NGLY1 and RNase B deglycosylation assay

FLAG-tagged human NGLY1 was overexpressed by the transduction of the pLenti expression vector that carries a Myc-DDK-tagged-human NGLY1 open reading frame in HEK293T cells. Anti-FLAG magnetic beads (OriGene Technologies, Rockville, MD) were used to react with the lysate of HEK293T cells overnight at 4°C in the presence of pan-protease inhibitors to purify the FLAG-tagged NGLY1. The magnetic beads were thoroughly washed using 0.5% Tween 20 in PBS for three times to minimize non-specific binding. The enrichment of recombinant human NGLY1 was checked by western blotting of NGLY1 in the pull-down fraction. To perform RNase B deglycosylation assays, purified human NGLY1 on an equal volume of magnetic beads was incubated with PBS containing each of the indicated small molecules and control vehicle (DMSO) at 37°C for 2 hours. The magnetic beads were then collected and resuspended in PBS containing 0.05% NP-40. Each magnetic bead suspension was mixed with 1ug RNase B (Millipore Sigma, St. Louis, MO) that was pre-denatured using 5mM DTT in the presence of 8M urea at 42°C for 1 hour followed by the treatment of 25mM iodoacetamide at room temperature for 1 hour and buffer exchange into PBS using Zeba 7K MWCO spin columns (Thermo Fisher Scientific, Carlsbad, CA). The mixtures of magnetic bead suspension and denatured RNase B were left at 37°C for 16 hours. The proteins in each mixture were resolved by SDS-PAGE and visualized using SYPRO Ruby gel stain (Thermo Fisher Scientific, Carlsbad, CA).

Proteomics Analysis

Cell samples were rinsed with PBS, harvested and snap-frozen. Each cell sample were mixed with 100μl of resuspension buffer containing 8M urea/1% (w/v) SDS/100 mM NH_4HCO_3 and 1% protease inhibitor cocktail (Millipore Sigma, St. Louis, MO) and subsequently sonicated for 5 minutes on ice. Reduction was performed by adding 5μl of 1M DTT and incubated for 1 hour at 30°C. Cell debris was removed by centrifugation at 14,000 x g for 5 minutes at room temperature. Vivacon spin column (30 kDa MWCO; Sartorius, Göttingen Germany) was washed using 100 µL of 8 M urea/100 mM NH_4HCO_3 and spun for 10 min at 14,000 x g at room temperature. After reduction, a reduced protein sample was transferred into a washed spin column and spun as described above. The spin column was washed once by adding 8M urea/100 mM NH_4HCO_3 and spinning for 10 min at 14,000 x g at room temperature. One hundred microliters of 55mM IAA/100mM NH_4HCO_3 was added to the spin column and allowed to

incubate with reduced proteins at room temperature for 20 minutes in the dark. IAA was removed by centrifugation and the spin column was washed twice with 8M urea/100mM NH_4HCO_3, followed by twice with 100µL of 50mM NH_4HCO_3. One hundred microliters of trypsin solution containing 5µg of trypsin made in 10mM NH_4HCO_3 was added to the spin column and allowed to incubate at 37°C overnight. After incubation, the collection tube was replaced with a new one and 50µL of 1% (v/v) formic acid added into the spin column and spun at 14,000 x g for 10 min. This step was repeated once. Flow through containing digested peptides was transferred to an HPLC vial and allowed to dry to completeness in a speed vacuum system. Digested peptides were resuspended in 100µL of 3% (v/v) ACN and peptide concentration was measured on a NanoDrop™ 2000/2000c Spectrophotometer (Thermo Fisher Scientific, Carlsbad, CA) at 205nm wavelength. All samples were acidified to final concentration of 0.1% (v/v) trifluoroacetic acid.

LC-MS/MS of digested proteins was performed using an Ultimate 3000 nano-flow system (Thermo Fisher Scientific, Carlsbad, CA) coupled to a LTQ XL Orbitrap ETD MS instrument (Thermo Fisher Scientific, Carlsbad, CA). Three biological and two technical replicates per sample were performed and randomly introduced into the LC system to minimize biological and technical variability introduced by the LC-MS system. One microliter of digested peptides (equivalent to 2µg) was drawn into a 1µl sample loop at 300nl/min flow rate using buffer A (2% (v/v) ACN/0.1% (v/v) FA) and sample directly flow from sample loop onto a trapping column (Acclaim PepMap100, C18, pore size 100Å, particle size 3µm, 75µm ID × 2 cm length; Thermo Fisher Scientific, Carlsbad, CA) and a Acclaim PepMap RSLC column (C18, pore size 100Å, particle size 2µm, 75µm internal diameter × 15cm length; Thermo Fisher Scientific, Carlsbad, CA). Peptide separation started after 15 minutes using a linear gradient of 5 to 45% (v/v) buffer B (80% (v/v) ACN/0.1% (v/v) FA) over 90 min, wash step of 90% buffer B for 10 minutes before column equilibration for 20 minutes in 5% buffer B. A total of 120 min of chromatographic time. LC and MS acquisition were controlled by Xcalibur version 2.1 (Thermo Fisher Scientific, Carlsbad, CA). The LTQ XL Orbitrap MS was operated in the data-dependent mode and spectra were acquired in positive mode in full MS scans in the mass range of 300 to 2000 *m/z* at a resolution of 60000 in the FT mode. The ten most intense precursor ions were then selected for isolation and subjected to CID fragmentation using a dynamic exclusion of 5 seconds. Dynamic exclusion criteria included a minimum relative signal intensity of 1000, and

≥2 positive charge state. An isolation width of 3.0 *m/z* was used with a normalized collision energy 35.

Spectra were analyzed using the MaxQuant software (version 1.5.3.17) with the Andromeda search engine [1] against the most recent version of UniProt human database. The standard Orbitrap settings in MaxQuant were used with a MS mass error tolerance of 20 ppm and MS/MS mass error tolerance of 0.5 Da. The variable modification of oxidation of methionine and HexNAc of asparagine, and the fixed modification of carbamidomethyl of cysteines were specified, with the digestion enzyme specified as trypsin. LFQ was activated with minimum ratio count of 2 and allowed match between runs as well as unidentified features. The LC-MS/MS runs were normalized according to the least overall proteome variation where majority of the proteins do not change between the samples. The false discovery rate (FDR) was set to 5% for both proteins and peptides, with a minimum peptide length of 7 amino acids. Only unique and razor peptides were used when reporting protein identifications.

Procedures of Chemical Synthesis and Characterization

Synthesis and Purification. Air sensitive reactions were carried out under a nitrogen atmosphere (Airgas Catalog No. NI UHP300). The following solvents were employed for chemical reactions: dichloromethane (99.9%, Extra Dry, AcroSeal™, Acros Organics Catalog No. 610300010), *N*,*N*-dimethylformamide (Anhydrous, 99.8%, packaged under Argon in resealable ChemSeal™ bottles, Catalog No. 43997) and ethyl alcohol (Absolute, anhydrous, ACS/USP grade, Pharmco-AAPER Catalog No. 111000200). The following solvents were employed for compound extractions: ethyl acetate (Certified ACS grade, Fisher Chemical Catalog No. E145-20) and dichloromethane (Not Stabilized, HPLC grade, Fisher Chemical Catalog No. D150-4). Saturated aqueous $NaHCO_3$ was prepared from deionized water and sodium bicarbonate (Reagent grade, Fisher Chemical Catalog No. S25533B). Brine was prepared from deionized water and sodium chloride (Reagent grade, Fisher Chemical Catalog No. S25541B). Organic extracts were dried over anhydrous sodium sulfate (Lab grade, Fisher Chemical Catalog No. S25568A). Thin layer chromatography (TLC) was conducted on glass plates coated with Silica Gel 60 F_{254} from Millipore Sigma (Catalog No. 1057150001). Normal phase flash chromatography was carried out on either a CombiFlash® EZ Prep or CombiFlash® Rf+ automated flash chromatography system, both from Teledyne ISCO. Normal phase flash

chromatography was carried out using Redi*Sep*® Rf normal phase disposable flash columns (40-60 micron) from Teledyne ISCO (Catalog Nos. 69-2203-304, 69-2203-312, 69-2203-324, 69-2203-340, 69-2203-380, and 69-2203-320). The following solvents were employed for TLC and normal phase chromatography: hexanes (Certified ACS grade, Fisher Chemical Catalog No. H292-20), ethyl acetate (Certified ACS grade, Fisher Chemical Catalog No. E145-20), dichloromethane (Not Stabilized, HPLC grade, Fisher Chemical Catalog No. D150-4), and methanol (HPLC grade, Fisher Chemical, Catalog No. A452-4). Reverse phase chromatography was carried out on a CombiFlash® EZ Prep automated flash chromatography system using a Redi*Sep*® Rf C18 column from Teledyne ISCO (Catalog No. 69-2203-413). Reverse phase preparative HPLC was carried out on a CombiFlash® EZ Prep automated flash chromatography system equipped with a Redi*Sep*® Prep C18 10 x 250 mm, 100Å, 5 μm HPLC preparative column from Teledyne ISCO (Catalog No. 692203809). The following solvents were employed for reverse phase chromatography: acetonitrile (HPLC grade, Fisher Chemical Catalog No. A998SK-4) and water purified using a Milli-Q® Advantage A10 Water Purification System from Millipore Sigma.

Characterization. All NMR spectra were recorded on a 300 MHz Bruker Fourier 300HD NMR spectrometer equipped with a dual ^{1}H and ^{13}C probe with Z-Gradient and automatic tuning and matching, full computer control of all shims with TopShim™, 24-sample SampleCase™ automation system, and TopSpin™ software. All NMR samples were prepared with either methyl sulfoxide-d_6 with 0.03% TMS, 99.8 atom % D, Acros Organics Catalog No. 360000100) or chloroform-d with 0.03% TMS, 99.8+ atom % D, Acros Organics Catalog No. 209561000). ^{1}H and ^{13}C chemical shifts are reported in δ values in ppm downfield with tetramethylsilane (TMS) as the internal standard. Data are reported as follows: chemical shift, multiplicity (s = singlet, d = doublet, t = triplet, q = quartet, b = broad, m = multiplet), integration, coupling constant (Hz). High resolution mass spectrometry was conducted on an Agilent 6230 Accurate-Mass Time-of-Flight (TOF) LC/MS with ESI source equipped with MassHunter Walkup software. MS parameters were as follows: fragmentor: 175 V, capillary voltage: 3500 V, nebulizer pressure: 35 psig, drying gas flow: 11 L/min, drying gas temperature: 325 ºC. Samples were introduced via an Agilent 1260 Infinity UHPLC comprised of a G4225A HiP Degasser, G1312B binary pump, G1367E ALS, G1316A TCC, and G1315C DAD VL+ with a 5 μL semi-micro flow cell with a 6 mm path length. UV absorption was observed at 220 nm and 254 nm

with a 4 nm bandwidth. Column: Agilent Zorbax SB-C18, Rapid Resolution HT, 1.8 µm, 2.1 x 50 mm. Gradient conditions: Hold at 5% CH_3CN in H_2O (0.1% formic acid) for 1.0 min, 5% to 95% CH_3CN in H_2O (0.1% formic acid) over 5 min, hold at 95% CH_3CN in H_2O (0.1% formic acid) for 1.0 min, 0.5 mL/min. All analogs were at least 95% pure according to these analytical methods.

Synthesis of Analogs. Analogs were prepared according to the scheme pictured below.

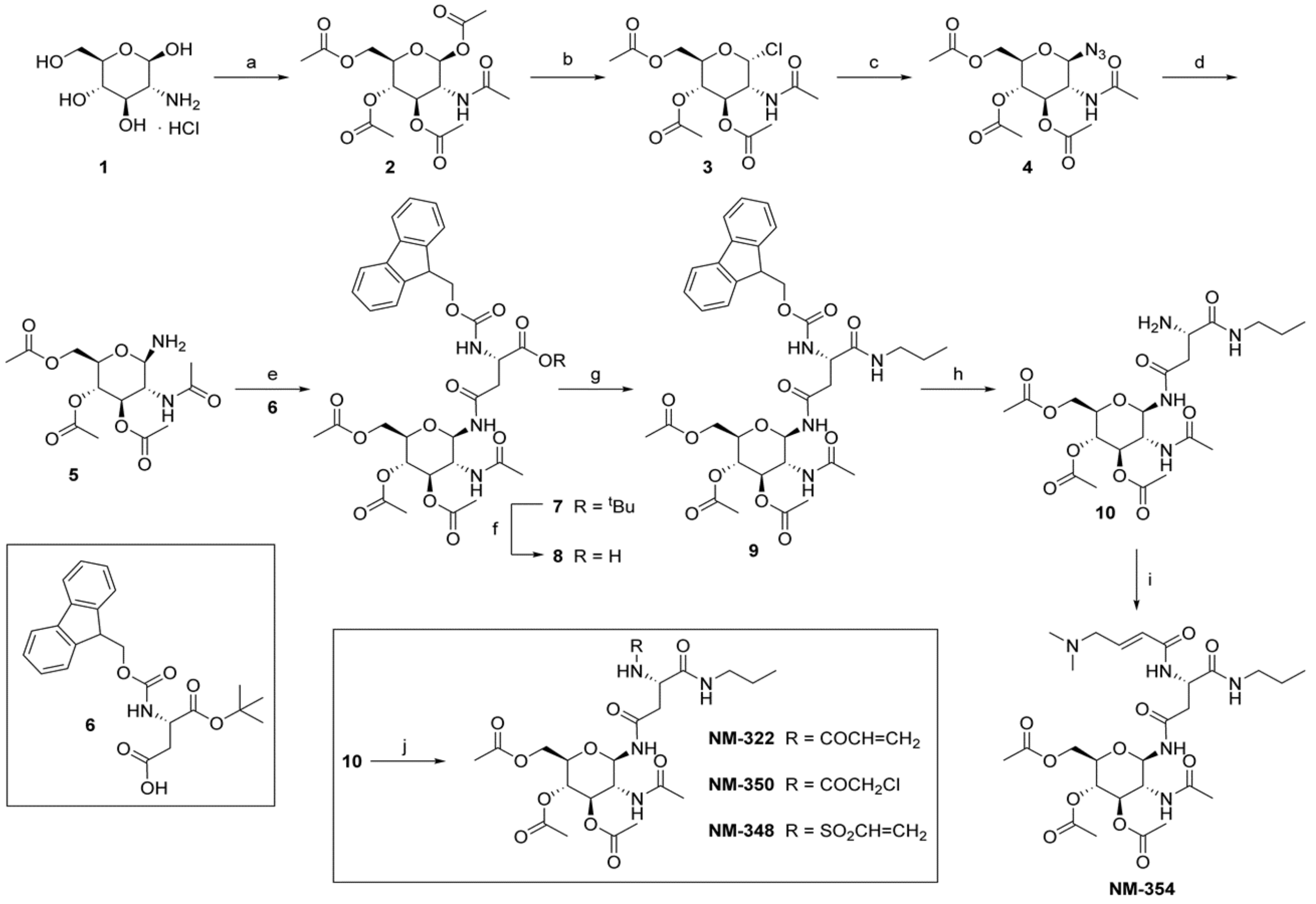

Reagents and Conditions: (a) acetic anhydride, pyridine, 82%; (b) HCl(g), acetic anhydride, 0 ºC to r.t., 65%; (c) (n-Bu)$_4NHSO_4$, NaN_3, CH_2Cl_2, sat'd aq. $NaHCO_3$, 89%; (d) HCO_2NH_4, 10% Pd/C, EtOH; (e) DIEA, HATU, CH_2Cl_2, 68% over 2 steps; (f) F_3CCO_2H, CH_2Cl_2, 0 ºC to r.t., 86%; (g) n-PrNH_2, DIEA, HATU, CH_2Cl_2; (h) piperidine, DMF; (i) 4-(dimethylamino)but-2-enoic acid hydrochloride, DIEA, HATU, CH_2Cl_2, 30%; (j) ClC(O)CH=CH_2 (for NM-322), ClC(O)CH_2Cl (for NM-350), or $ClSO_2CH_2CH_2Cl$ (for NM-348), DIEA, CH_2Cl_2, 0 ºC to r.t., 20-30%.

(2*S*,3*R*,4*R*,5*S*,6*R*)-3-Acetamido-6-(acetoxymethyl)tetrahydro-2*H*-pyran-2,4,5-triyl triacetate (2)

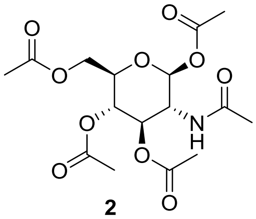

2

According to the method previously described [2], D-(+)-Glucosamine hydrochloride (USP grade, Chem-Impex Catalog No. 01450) (1.00 g, 4.64 mmol), pyridine (Anhydrous, DriSolv®, Millipore Sigma Catalog No. PX2012) (10 mL), and acetic anhydride (Certified ACS grade, Fisher Chemical Catalog No. A10-500) (2.62 mL, 27.9 mmol) were placed in a round bottom flask. The mixture was stirred at room temperature for 12 h. The reaction was monitored by TLC. After completion of the reaction, cold water was added and the resulting solution was extracted with ethyl acetate. The organic layer was washed with saturated aqueous $NaHCO_3$, brine, dried over anhydrous Na_2SO_4, and concentrated *in vacuo*. The crude product was purified by flash chromatography using hexane and ethyl acetate as eluent and obtained as a white solid (1.48 g, 82%): ^{1}H NMR (300 MHz, $CDCl_3$) δ 6.18 (d, *J* = 3.6 Hz, 1H), 5.54 (d, *J* = 8.9 Hz, 1H), 5.31 – 5.12 (m, 2H), 4.49 (td, *J* = 9.7, 9.7, 3.8 Hz, 1H), 4.26 (dd, *J* = 12.4, 4.0 Hz, 1H), 4.09 – 3.98 (m, 2H), 2.20 (s, 3H), 2.10 (s, 3H), 2.06 (s, 3H), 2.05 (s, 3H), 1.95 (s, 3H); ^{13}C NMR (75 MHz, $CDCl_3$) δ 171.64, 170.68, 169.97, 169.09, 168.65, 90.65, 70.61, 69.67, 67.46, 61.51, 50.99, 23.02, 20.93, 20.70, 20.69, 20.56.

(2*R*,3*S*,4*R*,5*R*,6*R*)-5-Acetamido-2-(acetoxymethyl)-6-chlorotetrahydro-2*H*-pyran-3,4-diyl diacetate (3)

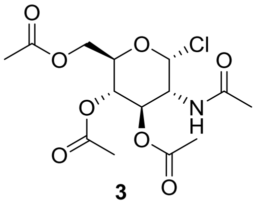

3

According to the method previously described [3], A solution of **2** (1.00 g, 2.73 mmol) in acetic anhydride (Certified ACS grade, Fisher Chemical Catalog No. A10-500) (10 mL) was cooled to

0 ºC and HCl (g) was added until the solution was saturated. The HCl(g) was generated by the slow dropwise addition of concentrated sulfuric acid (Fisher Chemical Catalog No. S25597) to sodium chloride (Reagent grade, Fisher Chemical Catalog No. S25541B) in a separate flask and transferred via tubing and needle to the reaction flask. The reaction mixture was allowed to warm to room temperature and stirred for 2 days. Upon completion of the reaction, the solvent was partially removed *in vacuo*. Water (25 mL) was added, and the solution was extracted with ethyl acetate (2x). The combined organic phases were washed with saturated aqueous $NaHCO_3$ and brine, dried over anhydrous Na_2SO_4, and concentrated *in vacuo*. The crude product was purified by flash chromatography using hexane and ethyl acetate as eluent and obtained as white solid (0.650 g, 65%): ^{1}H NMR (300 MHz, $CDCl_3$) δ 6.19 (d, *J* = 3.7 Hz, 1H), 5.80 (d, *J* = 8.7 Hz, 1H), 5.41 – 5.15 (m, 2H), 4.54 (ddd, *J* = 10.5, 8.8, 3.7 Hz, 1H), 4.35 – 4.22 (m, 2H), 4.19 – 4.08 (m, 1H), 2.11 (s, 3H), 2.06 (s, 3H), 2.06 (s, 3H), 1.99 (s, 3H); ^{13}C NMR (75 MHz, $CDCl_3$) δ 171.40, 170.55, 170.15, 169.12, 93.65, 70.88, 70.10, 66.99, 61.14, 53.44, 23.04, 20.67, 20.54.

(2*R*,3*S*,4*R*,5*R*,6*R*)-5-Acetamido-2-(acetoxymethyl)-6-azidotetrahydro-2*H*-pyran-3,4-diyl diacetate (4)

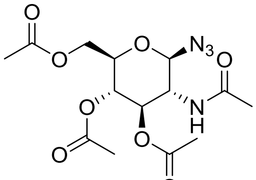

4

According to the previously described methods [4, 5], to a solution of **3** (1.97 g, 5.39 mmol), tetrabutylammonium hydrogen sulfate (TCI America Catalog No. T0835) (1.83 g, 5.39 mmol) and sodium azide (99%, extra pure, Acros Organics Catalog No. 190381000) (1.75 g, 26.9 mmol) in dichloromethane (19.7 mL) was added saturated aqueous $NaHCO_3$ (19.7 mL). The mixture was vigorously stirred at room temperature for 2-3 h, and the progress of reaction was monitored by TLC. After completion of the reaction, ethyl acetate (~200 mL) was added. The organic phase was separated, washed with saturated aqueous $NaHCO_3$, water, and brine. The organic phase was dried over anhydrous Na_2SO_4 and concentrated *in vacuo*. The crude product was purified by flash chromatography using hexane and ethyl acetate as eluent, dried, and obtained as white solid (1.78

g, 89%). Spectral data (^{1}H and ^{13}C NMR) were found in accordance with those previously published [4].

(2*R*,3*S*,4*R*,5*R*,6*R*)-5-Acetamido-2-(acetoxymethyl)-6-aminotetrahydro-2*H*-pyran-3,4-diyl diacetate (5)

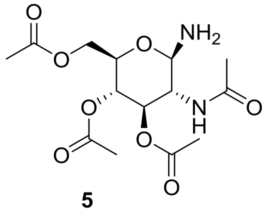

5

Ammonium formate (Sigma-Aldrich Catalog No. 156264) (139 mg, 2.20 mmol) and 10% palladium on carbon (Type 487, dry, Alfa Aesar Catalog No. A12012) (118 mg) were added to a solution of compound **4** (328 mg, 0.881 mmol) in dry ethanol (8 mL) and sealed in screw cap vial. The mixture was allowed to stir for 2 h at room temperature. After completion of reaction, methanol (5 mL) was added to the reaction mixture, and it was filtered through a syringe filter (0.2 μm). The filtrate was concentrated *in vacuo* and used directly in the next step without further purification. Spectral data (^{1}H and ^{13}C NMR) were found in accordance with those previously published [4].

(2*R*,3*S*,4*R*,5*R*,6*R*)-6-((*S*)-3-((((9*H*-Fluoren-9-yl)methoxy)carbonyl)amino)-4-(tert-butoxy)-4-oxobutanamido)-5-acetamido-2-(acetoxymethyl)tetrahydro-2*H*-pyran-3,4-diyl diacetate (7)

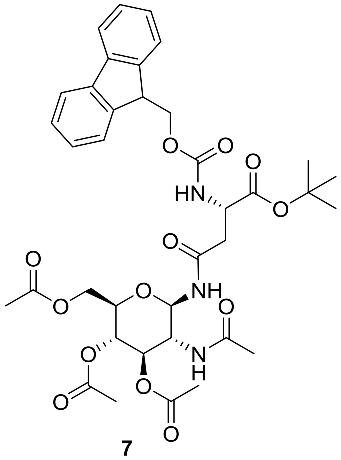

7

N,*N'*-Diisopropylethylamine (DIEA) (Chem-Impex Catalog No. 00141) (0.295 mL, 1.69 mmol) was added to a mixture of amino acid **6** (351 mg, 0.853 mmol) and O-(7-Aza-1H-benzotriazol-1-yl)-*N*,*N*,*N'*,*N'*-tetramethyluronium hexafluorophosphate (HATU) (Matrix Scientific Catalog No. 067222) (643 mg, 1.69 mmol) in dichloromethane (5 mL) under a N_2 atmosphere, and the reaction mixture was allowed to stir at room temperature. After 15 min, a solution of compound **5** (291 mg, 0.840 mmol) in dichloromethane (4 mL) was added, and the reaction mixture was stirred for an additional 12 h at room temperature. The progress of the reaction was monitored by TLC and LCMS. After completion of the reaction, water (10 mL) was added, and the mixture was extracted with dichloromethane (2x). The combined organic layers were dried over anhydrous Na_2SO_4 and concentrated *in vacuo*. The crude product was purified by flash chromatography using dichloromethane and methanol as eluent and obtained as a yellow solid (850 mg, 68%). Spectral data (^{1}H and ^{13}C NMR) were found in accordance with those previously published [4].

***N*^{2}-(((9*H*-fluoren-9-yl)methoxy)carbonyl)-*N*^{4}-((2*R*,3*R*,4*R*,5*S*,6*R*)-3-acetamido-4,5-diacetoxy-6-(acetoxymethyl)tetrahydro-2*H*-pyran-2-yl)-L-asparagine (8)**

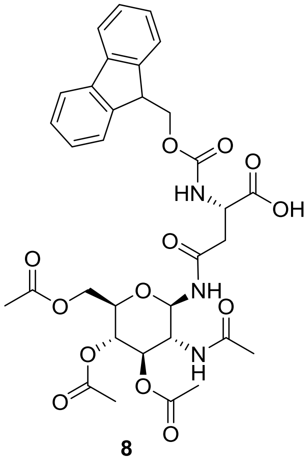

8

Compound **7** (1.05 g, 1.42 mmol) was dissolved in dichloromethane (500 μL) with stirring and cooled in an ice bath. 95% Trifluoracetic acid (Alfa Aesar Catalog No. A12198) in dichloromethane (5 mL) was added, and the reaction mixture was stirred at room temperature for 2h. The progress of the reaction was monitored by TLC and LCMS. After completion of the reaction, solvent was removed *in vacuo,* and the crude product was purified by reverse phase chromatography using acetonitrile and water as eluent. The product was obtained as as a white

solid (835 mg, 86%). Spectral data (^{1}H and ^{13}C NMR) were found in accordance with those previously published [4].

(2*R*,3*S*,4*R*,5*R*,6*R*)-6-((*S*)-3-((((9*H*-Fluoren-9-yl)methoxy)carbonyl)amino)-4-oxo-4-(propylamino)butanamido)-5-acetamido-2-(acetoxymethyl)tetrahydro-2*H*-pyran-3,4-diyl diacetate (9)

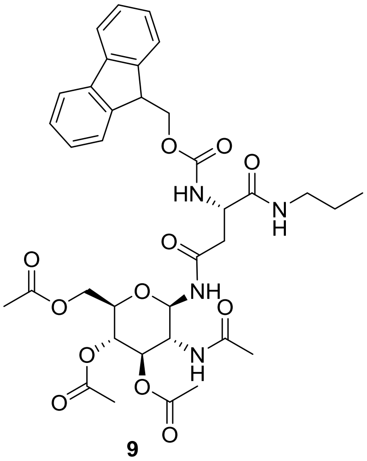

9

DIEA (Chem-Impex Catalog No. 00141) (141 µL, 0.878 mmol) was added to a mixture of compound **8** (300 mg, 0.439 mmol) and HATU (Matrix Scientific Catalog No. 067222) (335 mg, 0.878 mmol) in dichloromethane (10 mL) under a nitrogen atmosphere. The reaction mixture was allowed to stir at room temperature. After 15 mins, *n*-propylamine (Alfa Aesar Catalog No. 36635) (181 µL, 2.20 mmol) in dichloromethane (1 mL) was added. Stirring continued for 12-15h at room temperature. The progress of the reaction was monitored by TLC and LCMS. After completion of the reaction, solvent was removed *in vacuo*, and the crude product was used in the next step without any further purification.

(2*R*,3*S*,4*R*,5*R*,6*R*)-5-Acetamido-2-(acetoxymethyl)-6-((*S*)-3-amino-4-oxo-4-(propylamino)butanamido)tetrahydro-2*H*-pyran-3,4-diyl diacetate (10)

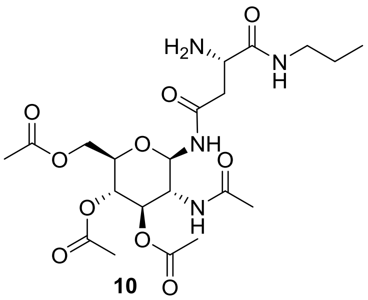

10

Compound **9** (300 mg, 0.413 mmol) was dissolved in 20% piperidine (Sigma-Aldrich Catalog No. 104094) solution in DMF (4 mL), and the solution was stirred for one hour at room temperature. After completion of the reaction as judged by LCMS, the solvent was removed *in vacuo*, and the crude product was purified by reverse phase chromatography using acetonitrile and water as eluent to afford the product as a white solid (175 mg, 84%): ^{1}H NMR (300 MHz, DMSO-d_6) δ 8.71 (d, *J* = 9.5 Hz, 1H), 8.00 – 7.79 (m, 2H), 5.12 (dd, *J* = 18.5, 9.0 Hz, 2H), 4.82 (t, *J* = 9.7, 1H), 4.26 – 4.11 (m, 1H), 4.02 – 3.76 (m, 3H), 3.51 – 3.37 (m, 1H), 3.34 – 3.22 (m, 1H), 3.01 (q, *J* = 7.0 Hz, 3H), 2.00 (s, 3H), 1.97 (s, 3H), 1.91 (s, 3H), 1.74 (s, 3H), 1.40 (q, *J* = 7.2 Hz, 2H), 0.83 (t, *J* = 7.4 3H).

FINAL ANALOGS

(2*R*,3*S*,4*R*,5*R*,6*R*)-5-acetamido-2-(acetoxymethyl)-6-((*S*)-3-acrylamido-4-oxo-4-(propylamino)butanamido)tetrahydro-2*H*-pyran-3,4-diyl diacetate (NM-322)

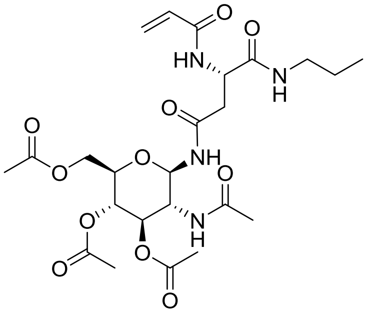

NM-322

A solution of acryloyl chloride (Alfa Aesar Catalog No. L10363) (9.68 µL, 0.119 mmol) in DCM (500 µL) was added dropwise via syringe to a mixture of compound **10** (50 mg, 0.099 mmol) and triethylamine (Acros Organics Catalog No. 157911000) (30 µL, 0.22 mmol) in dichloromethane (3 mL) in an ice bath. The mixture was allowed to warm to room temperature and stirred for an additional 3-4h. After completion of the reaction, the solvent was removed *in vacuo*, and the crude

compound was purified by preparative HPLC using acetonitrile and water as eluent to afford the product as a white solid (21 mg, 38%): ^{1}H NMR (300 MHz, DMSO-d$_6$) δ 8.51 (d, *J* = 9.5 Hz, 1H), 8.25 (d, *J* = 8.2 Hz, 1H), 7.88 (d, *J* = 9.1 Hz, 1H), 7.79 (t, *J* = 5.7 Hz, 1H), 6.27 (dd, *J* = 17.1, 10.1 Hz, 1H), 6.07 (dd, *J* = 17.1, 2.3 Hz, 1H), 5.58 (dd, *J* = 10.0, 2.3 Hz, 1H), 5.13 (dt, *J* = 19.9, 9.7 Hz, 2H), 4.81 (t, *J* = 9.7 Hz, 1H), 4.63 (q, *J* = 7.5 Hz, 1H), 4.17 (dd, *J* = 12.4, 4.2 Hz, 1H), 3.99 – 3.75 (m, 3H), 2.99 (q, *J* = 6.3 Hz, 2H), 2.59 (dd, *J* = 15.9, 6.2 Hz, 1H), 2.39 (dd, *J* = 16.0, 7.4 Hz, 1H), 1.99 (s, 3), 1.96 (s, 3H), 1.90 (s, 3H), 1.73 (s, 3H), 1.42 – 1.34 (m, 2H), 0.80 (t, *J* = 7.4, 3H); ^{13}C NMR (75 MHz, DMSO-d$_6$) δ 170.90, 170.51, 170.29, 169.97, 169.94, 169.79, 164.72, 132.08, 125.86, 78.37, 73.81, 72.67, 68.79, 62.28, 52.61, 50.63, 49.77, 37.89, 23.11, 22.68, 21.00, 20.88, 20.84, 11.74; HRMS, calc'd for $C_{24}H_{37}N_4O_{11}^+$ [M+H], 557.2453; found 557.2454.

(2*R*,3*S*,4*R*,5*R*,6*R*)-5-Acetamido-2-(acetoxymethyl)-6-((*S*)-3-(2-chloroacetamido)-4-oxo-4-(propylamino)butanamido)tetrahydro-2*H*-pyran-3,4-diyl diacetate (NM-350).

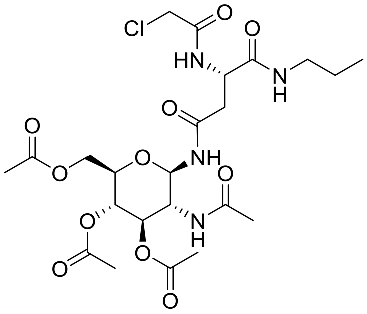

NM-350

A solution of chloracetyl chloride (Alfa Aesar Catalog No. A15846) (10.8 mg, 0.095 mmol) in DCM (500 µL) was added dropwise via syringe to a mixture of compound **10** (40 mg, 0.079 mmol) and triethylamine (Acros Organics Catalog No. 157911000) (25 µL, 0.18 mmol) in dichloromethane (3 mL) in an ice bath. The mixture was allowed to warm to room temperature and stirred for an additional 3-4h. After completion of the reaction, the solvent was removed *in vacuo*, and the crude compound was purified by preparative HPLC using acetonitrile and water as eluent to afford the product as a white solid (25 mg, 54%): ^{1}H NMR (300 MHz, DMSO-d$_6$) δ 8.53 (d, *J* = 9.4 Hz, 1H), 8.34 (d, *J* = 8.3 Hz, 1H), 7.88 (d, *J* = 9.3 Hz, 1H), 7.82 (t, *J* = 5.9 Hz, 1H), 5.13 (dt, *J* = 19.8, 9.7 Hz, 2H), 4.81 (t, *J* = 9.8 Hz, 1H), 4.54 (q, *J* = 7.0 Hz, 1H), 4.20 – 4.11 (m, 1H), 4.09 (s, 2H), 4.03 – 3.77 (m, 3H), 2.99 (q, *J* = 6.1 Hz, 2H), 2.57 (dd, *J* = 15.9, 5.9

Hz, 1H), 2.48 – 2.39 (m, 1H), 1.99 (s, 3H), 1.96 (s, 3H), 1.90 (s, 3H), 1.74 (s, 3H), 1.38 (h, *J* = 7.2 Hz, 2H), 0.81 (t, *J* = 7.4 Hz, 3H); ^{13}C NMR (75 MHz, DMSO-d$_6$) δ 170.49, 170.40, 170.38, 170.33, 169.97, 169.77, 166.00, 78.39, 73.85, 72.71, 68.82, 62.30, 52.57, 50.08, 43.11, 37.74, 23.08, 22.76, 22.68, 20.99, 20.87, 20.84, 11.74; HRMS, calc'd for $C_{23}H_{36}ClN_4O_{11}^+$ [M+H], 579.2064; found 579.2067.

(2*R*,3*S*,4*R*,5*R*,6*R*)-5-Acetamido-2-(acetoxymethyl)-6-((*S*)-4-oxo-4-(propylamino)-3-(vinylsulfonamido)butanamido)tetrahydro-2*H*-pyran-3,4-diyl diacetate (NM-348)

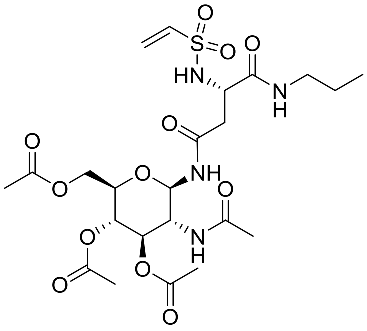

NM-348

A solution of 2-Chloroethanesulfonyl chloride (TCI America Catalog No. C1142) (14.5 mg, 0.089 mmol) in DCM (500 µL) was added dropwise via syringe to a mixture of compound **10** (45 mg, 0.089 mmol) and triethylamine (Acros Organics Catalog No. 157911000) (25 µL, 0.18 mmol) in dichloromethane (3 mL) in an ice bath. The mixture was allowed to warm to room temperature and stirred for an additional 3-4h. After completion of the reaction, the solvent was removed *in vacuo*, and the crude compound was purified by preparative HPLC using acetonitrile and water as eluent to afford the product as a white solid (27 mg, 50%): ^{1}H NMR (300 MHz, DMSO-d$_6$) δ 8.64 – 8.44 (m, 1H), 7.90 (d, *J* = 9.06 Hz, 1H), 7.83 (t, *J* = 5.80, 2H), 7.57 (s, 1H), 6.63 (dd, *J* = 16.5, 9.9 Hz, 1H), 6.10 – 5.77 (m, 2H), 5.26 – 5.03 (m, 2H), 4.82 (t, *J* = 9.8, 1H), 4.18 (dd, *J* = 12.3, 4.1 Hz, 1H), 4.10 – 3.72 (m, 4H), 2.99 (q, *J* = 6.5 Hz, 2H), 2.56 (d, *J* = 6.0 Hz, 1H), 2.46 – 2.31 (m, 1H), 2.00 (s, 3H), 1.97 (s, 3H), 1.91 (s, 3H), 1.76 (s, 3H), 1.41 (dt, *J* = 14.3, 7.4 Hz, 2H), 0.82 (t, *J* = 7.3 Hz, 2H); ^{13}C NMR (75 MHz, DMSO-d$_6$) δ 170.50, 170.13, 170.08, 170.02, 170.00, 169.77, 137.86, 125.71, 78.43, 73.77, 72.69, 68.84, 62.31, 53.30, 52.60, 52.04, 39.03, 23.12, 22.63, 20.99, 20.88, 20.84, 11.75; HRMS, calc'd for $C_{23}H_{37}N_4O_{12}S^+$ [M+H], 593.2123; found 593.2135.

(2*R*,3*S*,4*R*,5*R*,6*R*)-5-Acetamido-2-(acetoxymethyl)-6-((*S*)-3-((*E*)-4-(dimethylamino)but-2-enamido)-4-oxo-4-(propylamino)butanamido)tetrahydro-2*H*-pyran-3,4-diyl diacetate (NM-354)

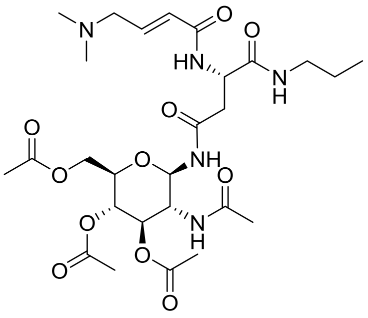

NM-354

DIEA (Chem-Impex Catalog No. 00141) (31 µL, 0.191 mmol) was added to the mixture of Compound **10** (48 mg, 0.0956 mmol) and HATU (Matrix Scientific Catalog No. 067222) (48 mg, 0.124 mmol) in dichloromethane (3 mL) under a nitrogen atmosphere and stirred for 15 min. A solution of 4-(dimethylamino)but-2-enoic acid hydrochloride (Ark Pharm Catalog No. AK-44120) (16 mg, 0.0965 mmol) in dichloromethane (2 mL) was added to the mixture, and it was stirred for an additional 10h. After completion of the reaction, the solvent was removed *in vacuo*, and the crude product was purified by preparative HPLC using acetonitrile and water as eluent to afford the product (17.2 mg, 30%): ^{1}H NMR (300 MHz, CD_3OD) δ 8.61 (d, *J* = 9.1 Hz, 1H), 8.44 (d, *J* = 8.0 Hz, 1H), 8.15 (d, *J* = 9.2 Hz, 1H), 7.95 (t, *J* = 5.6 Hz, 1H), 6.71 (dt, *J* = 14.7, 7.3 Hz, 1H), 6.40 (d, *J* = 15.3 Hz, 1H), 5.29 – 5.11 (m, 2H), 5.05 – 4.92 (m, 2H), 4.24 (dd, *J* = 12.4, 4.4 Hz, 1H), 4.12 – 3.85 (m, 4H), 3.85 – 3.79 (m, 1H), 3.20 – 3.04 (m, 2H), 2.90 (s, 6H), 2.81 – 2.56 (m, 2H), 2.02 (s, 3H), 2.00 (s, 3H), 1.98 (s, 3H), 1.87 (s, 3H), 1.51 (h, *J* = 7.2 Hz, 2H), 0.90 (t, *J* = 7.4 Hz, 3H); ^{13}C NMR (75 MHz, CD_3OD) δ 172.33, 171.10, 171.08, 170.86, 170.37, 169.90, 164.45, 132.06, 130.24, 78.22, 73.31, 73.26, 68.50, 61.88, 57.40, 52.76, 50.09, 41.84, 40.97, 37.16, 22.12, 21.34, 19.20, 19.17, 19.13, 10.26; HRMS, calc'd for $C_{27}H_{44}N_5O_{11}^+$ [M+H], 614.3032; found 614.3037.

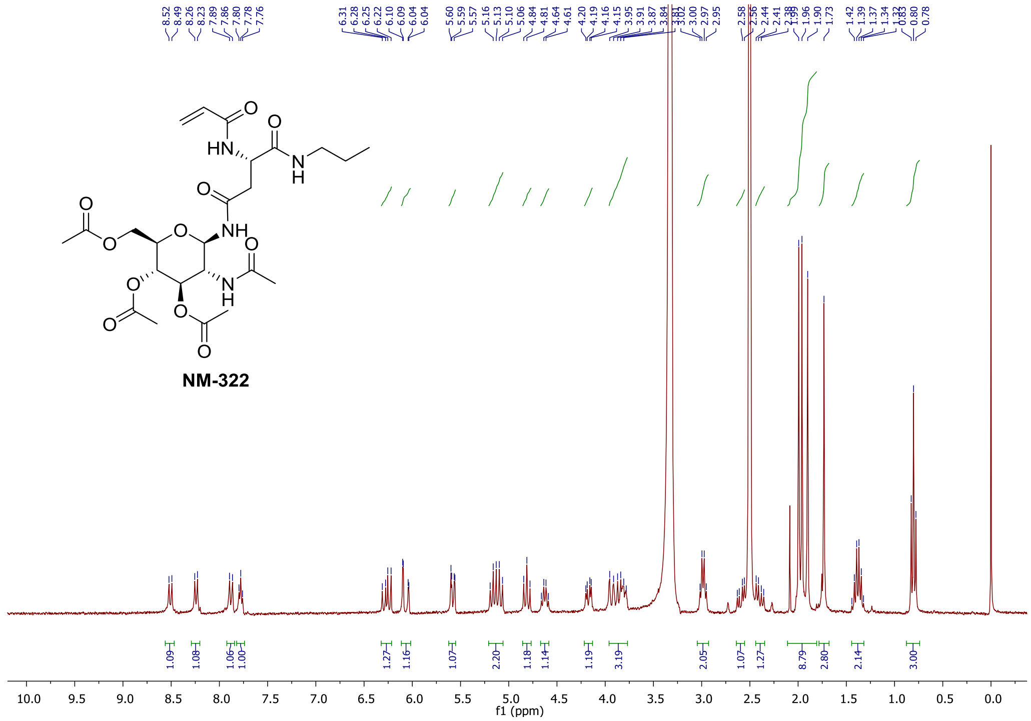

^1H NMR spectrum of compound **NM-322** (300 MHz, DMSO-d_6)

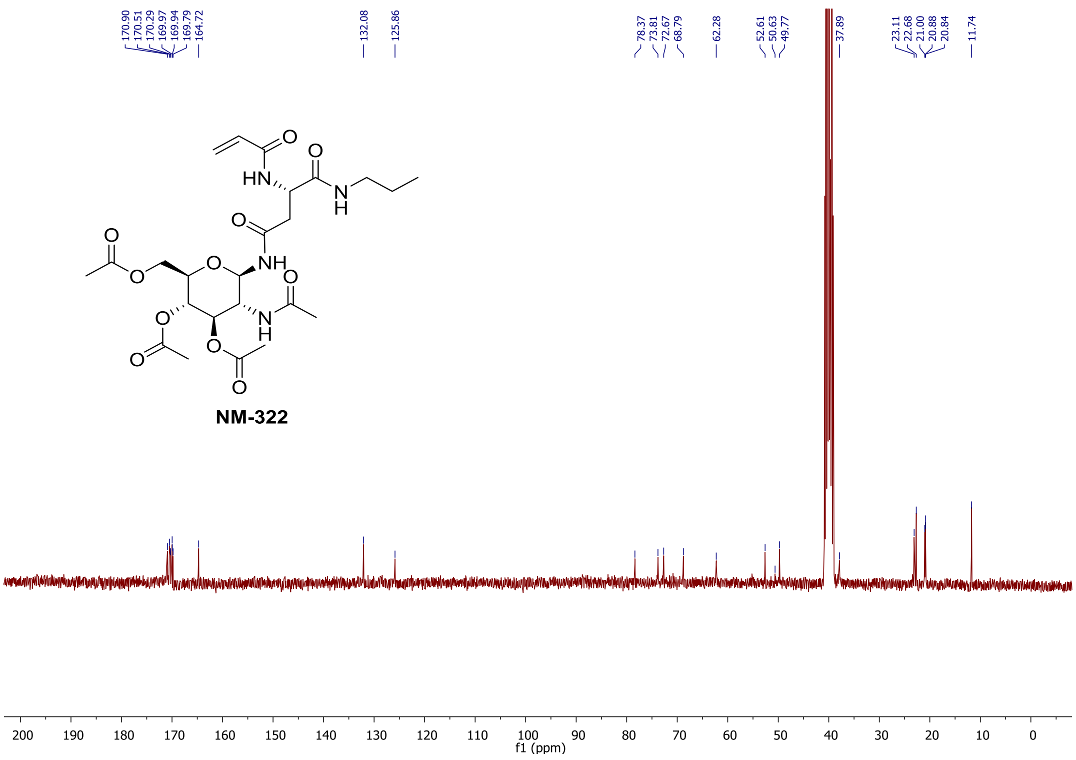

^{13}C NMR spectrum of compound **NM-322** (75 MHz, DMSO-d_6)

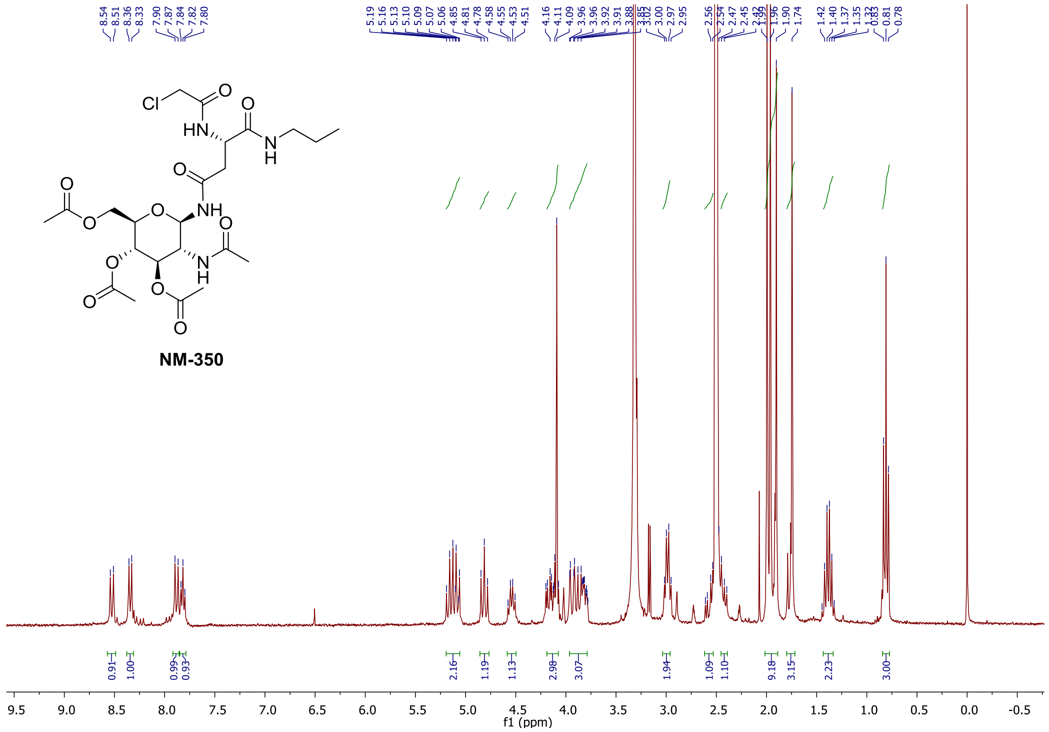

^{1}H NMR spectrum of compound **NM-350** (300 MHz, DMSO-d_6)

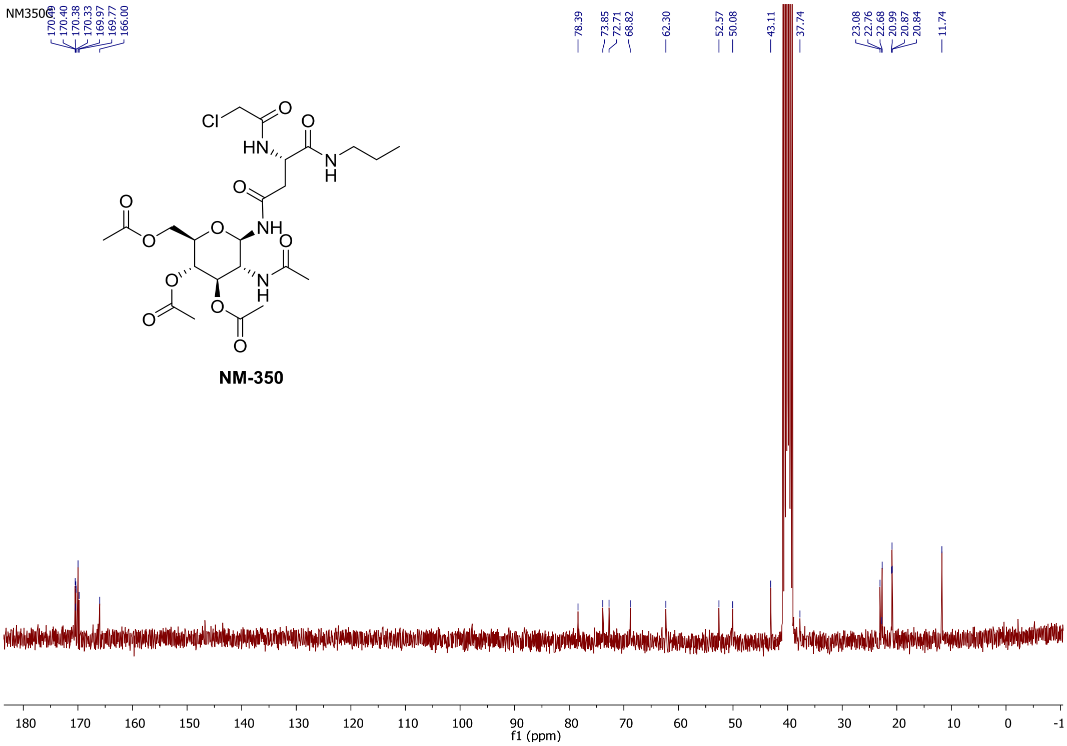

^{13}C NMR spectrum of compound **NM-350** (75 MHz, DMSO-d_6)

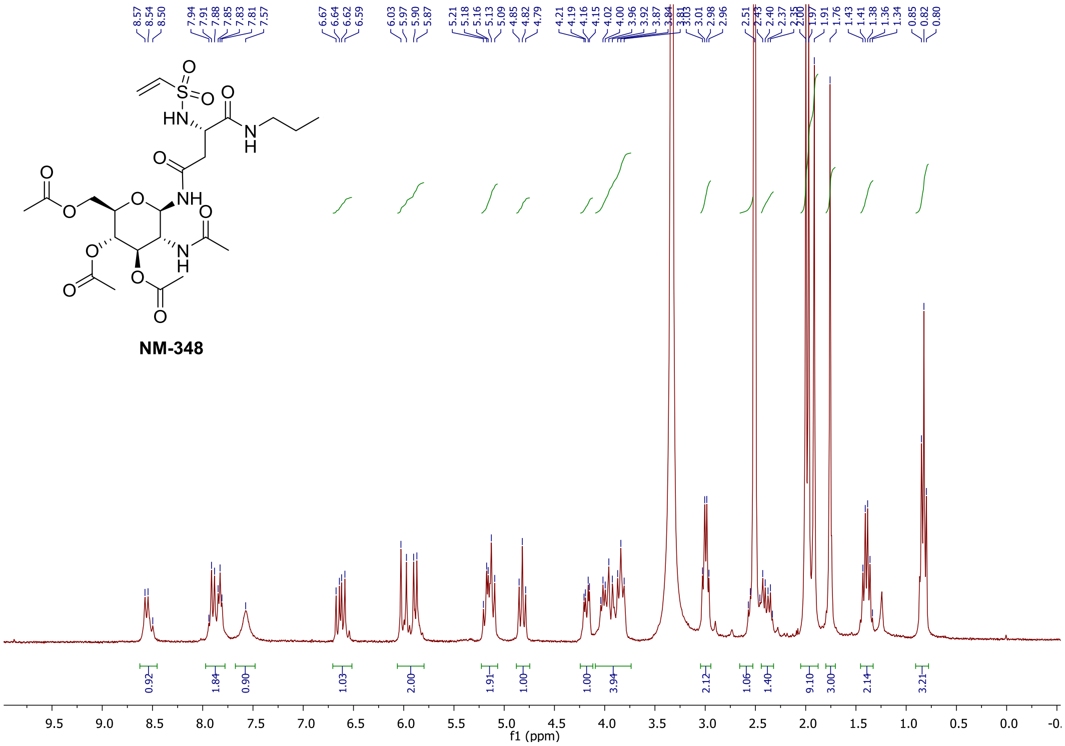

^{1}H NMR spectrum of compound **NM-348** (300 MHz, DMSO-d$_6$)

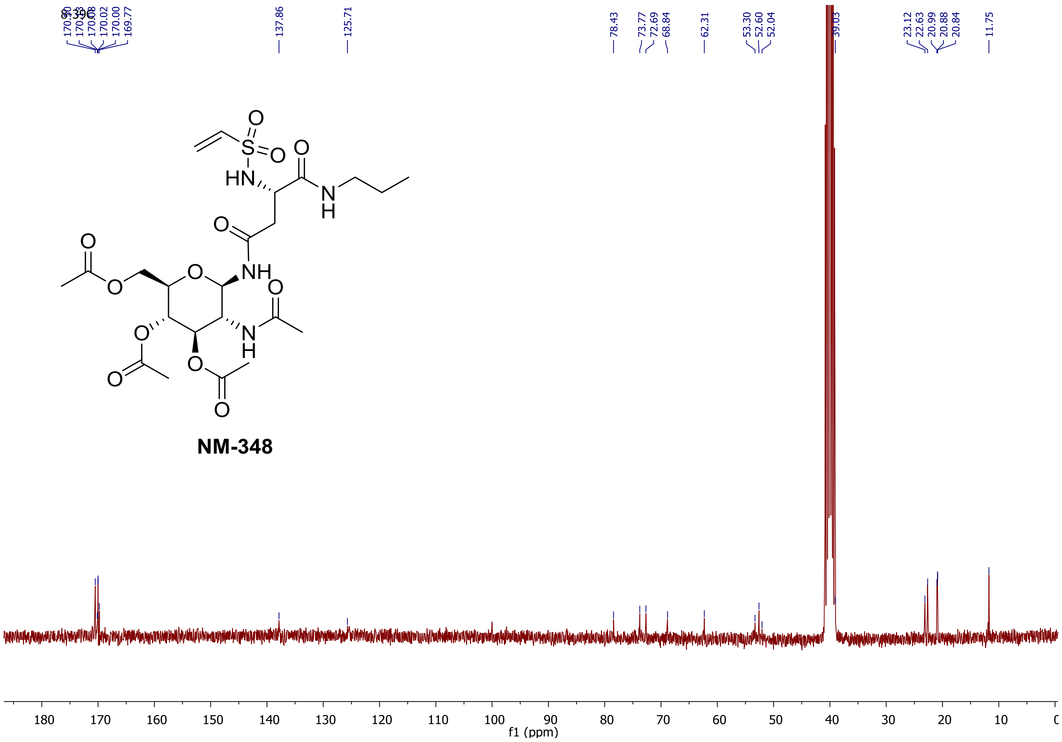

^{13}C NMR spectrum of compound **NM-348** (75 MHz, DMSO-d$_6$)

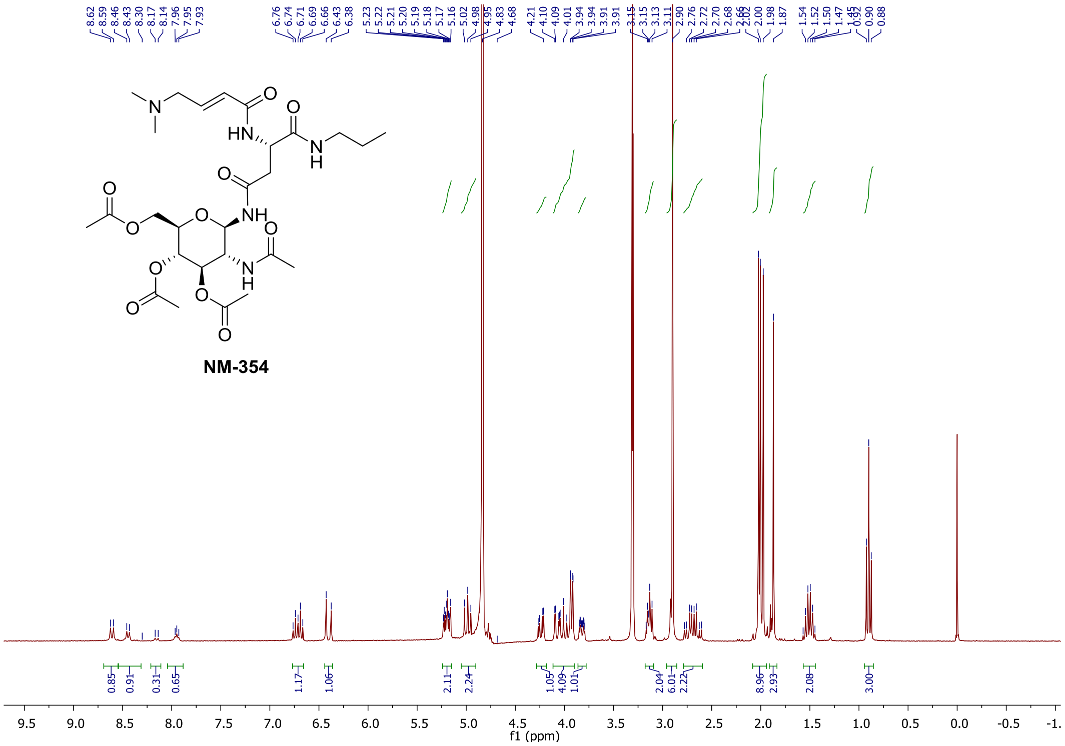

^{1}H NMR spectrum of compound **NM-354** (300 MHz, DMSO-d_6)

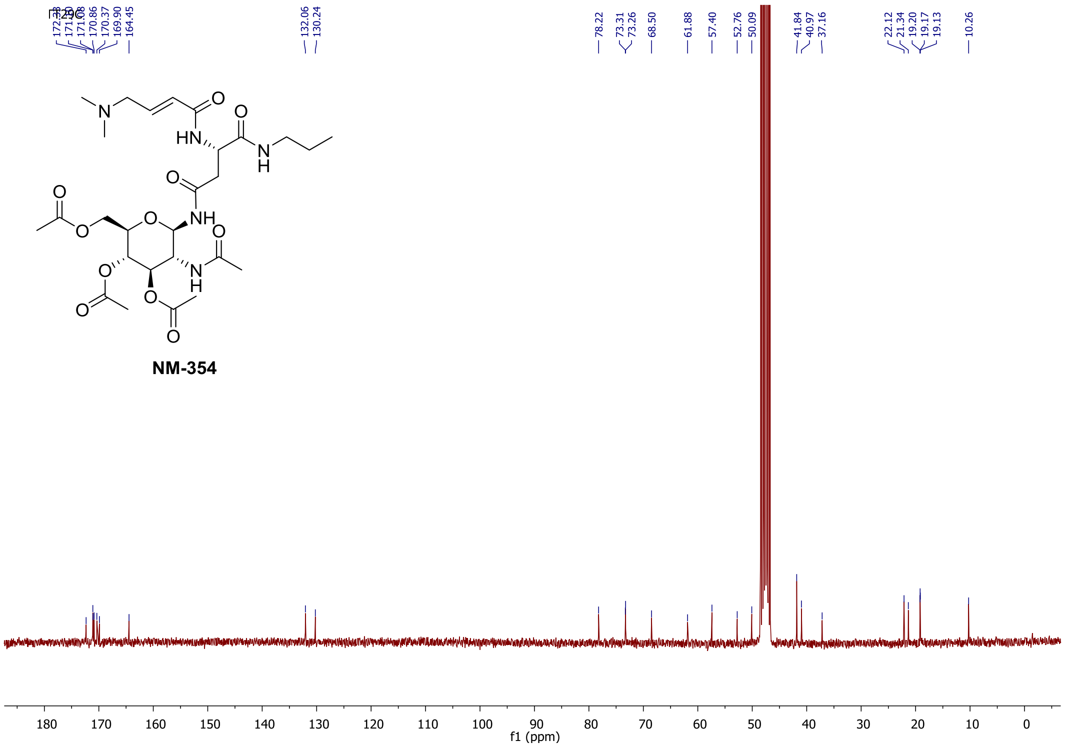

^{13}C NMR spectrum of compound **NM-354** (75 MHz, DMSO-d_6)

Computational Modeling

We used the SWISS-MODEL [6] and the crystallographic structure of the mouse NGLY1 (PDB code: 2F4M) as a template to build the structural homology model of human NGLY1 core domain. After the human homology model was obtained, Autodock tools [7] were used to prepare the receptor based on the homology model for compound docking. Each ligand (a compound) was also prepared using the Autodock tools with all single bonds of each ligand left rotatable and all amide bonds fixed in a trans position. The docking process was performed using Autodock Vina [8] with 30 as exhaustiveness to enable the generation of 20 binding poses for each ligand. The binding poses with distances of less than 6 Å between the sulfur of Cys309 of the human NGLY1 and the distal carbon involved in the double bond of the electrophilic replacement (R) group for each tested ligand were ranked based on their docking energies. For Z-VAD-fmk and NM-350, distances between the sulfur of Cys309 and the leaving groups (F and Cl) of two ligands for each binding pose were measured to select candidate poses for ranking. The selected poses with the lowest binding energies in ranking were considered the most favorable binding poses.

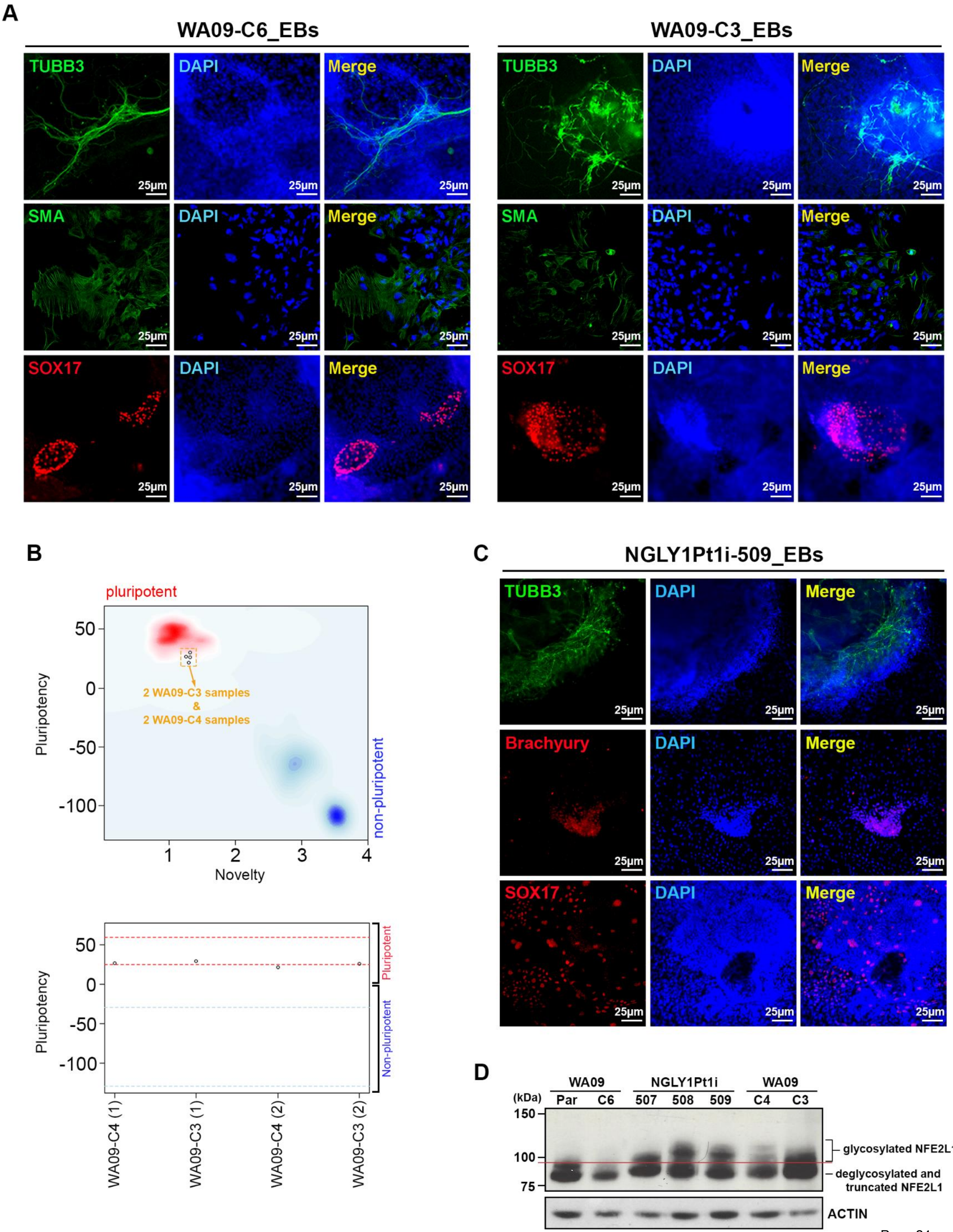
A
WA09-C6_EBs
WA09-C3_EBs
TUBB3
DAPI
Merge
SMA
SOX17
25μm
B
pluripotent
non-pluripotent
2 WA09-C3 samples
&
2 WA09-C4 samples
Pluripotency
Novelty
Pluripotent
Non-pluripotent
WA09-C4 (1)
WA09-C3 (1)
WA09-C4 (2)
WA09-C3 (2)
C
NGLY1Pt1i-509_EBs
Brachyury
D
WA09
NGLY1Pt1i
(kDa)
Par
C6
507
508
509
C4
C3
150
100
75
glycosylated NFE2L1
deglycosylated and truncated NFE2L1
ACTIN

Supplementary Figure S1. Cellular pluripotency tests and NFE2L1 expression in hPSCs without NGLY1 expression. (**A**) WA09-C6 (control) and WA09-C3 (NGLY1-KO) hESCs formed EBs containing differentiated cells that are associated with three germ-layer lineages (TUBB3: ectoderm marker, SMA: mesoderm marker, and SOX17: endoderm marker). *Left panel:* WA09-C6 EBs. *Right panel:* WA09-C3 EBs. (**B**) The Pluritest results of undifferentiated WA09-C3 and WA09-C4 hESCs that are two independent NGLY1-knockout subclones revealed that their transcriptomic features are highly similar to the transcriptomic features of hPSC samples included in the Pluritest database. (**C**) NGLY1-deficient patient-derived hiPSCs formed EBs containing differentiated cells that are associated with three germ-layer lineages (TUBB3: ectoderm marker, Brachyury: mesoderm marker, and SOX17: endoderm marker). (**D**) The electrophoretic mobility shift of NFE2L1 (migrating towards the location of 100 kDa or above) indicating its retention of *N*-glycans was detected in NGLY1-kockout WA09 hESCs (WA09-C3, WA09-C4 cells) and NGLY1 deficient patient-derived iPSCs (NGLY1Pt1i-507, NGLY1Pt1i-508 and NGLY1Pt1i-509 cells) treated with 20 µM bortezomib for 4 hours. *Par:* parental WA09 hESCs. *C6:* WA09-C6 cells.

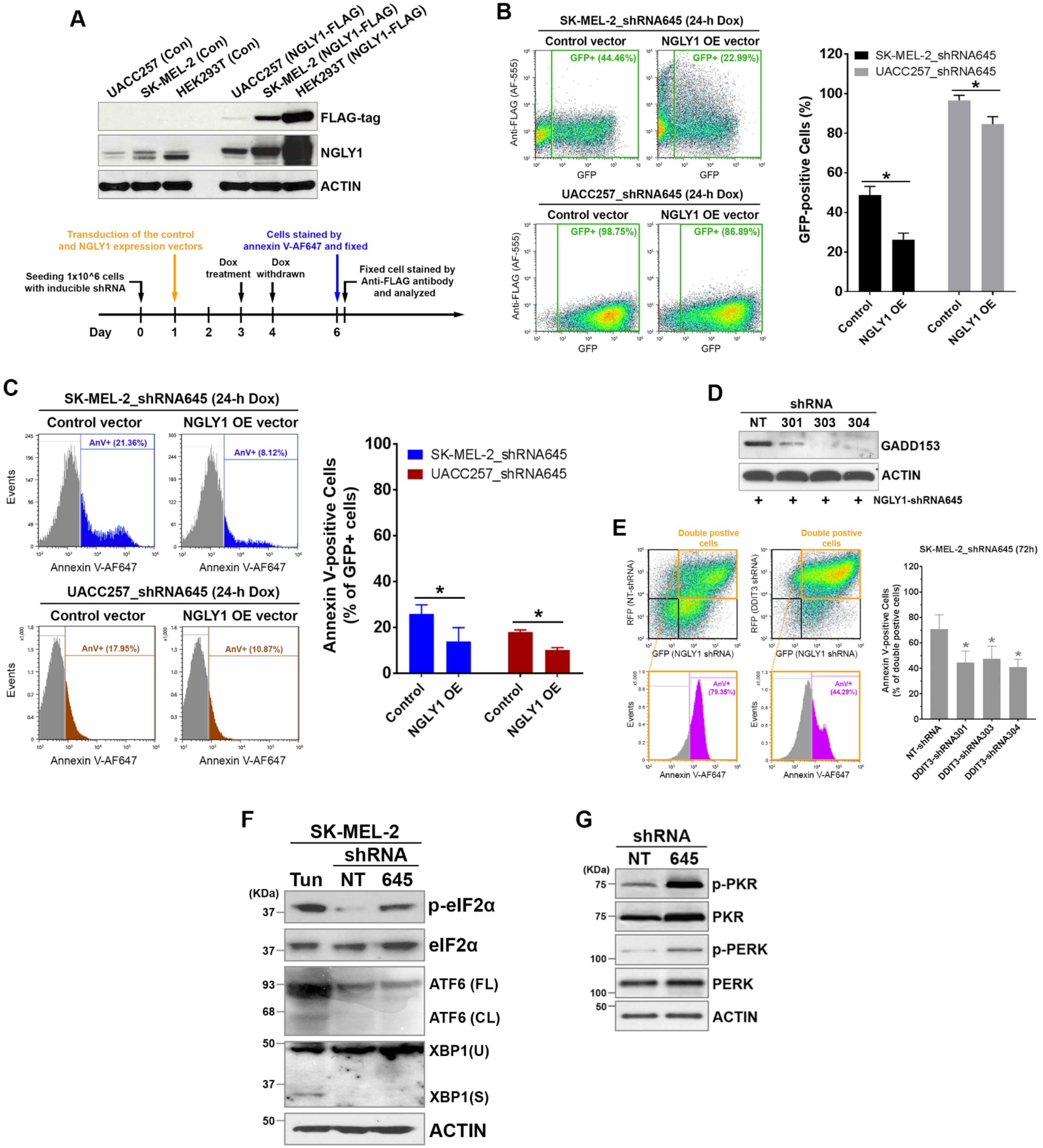
A
UACC257 (Con)
SK-MEL-2 (Con)
HEK293T (Con)
UACC257 (NGLY1-FLAG)
SK-MEL-2 (NGLY1-FLAG)
HEK293T (NGLY1-FLAG)
FLAG-tag
NGLY1
ACTIN
Transduction of the control and NGLY1 expression vectors
Cells stained by annexin V-AF647 and fixed
Seeding 1x10^6 cells with inducible shRNA
Dox treatment
Dox withdrawn
Fixed cell stained by Anti-FLAG antibody and analyzed
Day
0
1
2
3
4
6
B
SK-MEL-2_shRNA645 (24-h Dox)
Control vector
NGLY1 OE vector
GFP+ (44.46%)
GFP+ (22.99%)
Anti-FLAG (AF-555)
GFP
UACC257_shRNA645 (24-h Dox)
GFP+ (98.75%)
GFP+ (86.89%)
SK-MEL-2_shRNA645
UACC257_shRNA645
GFP-positive Cells (%)
Control
NGLY1 OE
C
SK-MEL-2_shRNA645 (24-h Dox)
Control vector
NGLY1 OE vector
AnV+ (21.36%)
AnV+ (8.12%)
Events
Annexin V-AF647
UACC257_shRNA645 (24-h Dox)
AnV+ (17.95%)
AnV+ (10.87%)
Annexin V-positive Cells (% of GFP+ cells)
SK-MEL-2_shRNA645
UACC257_shRNA645
Control
NGLY1 OE
D
shRNA
NT
301
303
304
GADD153
ACTIN
NGLY1-shRNA645
E
Double postive cells
RFP (NT-shRNA)
RFP (DDIT3 shRNA)
GFP (NGLY1 shRNA)
AnV+ (79.35%)
AnV+ (44.29%)
Events
Annexin V-AF647
SK-MEL-2_shRNA645 (72h)
Annexin V-positive Cells (% of double postive cells)
NT-shRNA
DDIT3-shRNA301
DDIT3-shRNA303
DDIT3-shRNA304
F
SK-MEL-2
shRNA
Tun
NT
645
(KDa)
p-eIF2α
eIF2α
ATF6 (FL)
ATF6 (CL)
XBP1(U)
XBP1(S)
ACTIN
G
shRNA
NT
645
(KDa)
p-PKR
PKR
p-PERK
PERK
ACTIN

Supplementary Figure S2. The attenuation of NGLY1 knockdown-induced apoptosis by NGLY1 overexpression and GADD153 knockdown in melanoma cells. (**A**) *Upper panel:* The overexpression of FLAG-tagged human NGLY1 in melanoma and HEK293T cells. *Lower panel:* The schematic illustration of testing the rescue effect of NGLY1 overexpression in melanoma cells with induced NGLY1 knockdown. (**B**) The overexpression of human NGLY1 attenuated the induced expression of ZsGreen (GFP) in melanoma cells, indicating that the expanded pool of NGLY1 transcripts effectively interfered with the induced ZsGreen-shRNA transcripts. *Left panel:* Dot plot representation of flow cytometry analysis in the cells. *Right panel:* The percentages of GFP-positive cells in the dox-induced melanoma cells that received the control and NGLY1 overexpression (NGLY1 OE) vectors. (**C**) The analysis of apoptotic (annexin V-stained) cells in the GFP-positive populations of the dox-induced melanoma cells that received the control and NGLY1 OE vectors. (**D**) The shRNA-mediated suppression of NGLY knockdown-induced GADD153 (DDIT3) in SK-MEL-2 cells. SK-MEL-2 cells with inducible NGLY1-shRNA645 were transduced with GADD153-targeting shRNA and subsequently treated with 2μM dox for 48 hours to induce the expression of NGLY1-targeting shRNA. Three independent shRNA sequences that target GADD153: shRNA301, shRNA303 and shRNA304. (**E**) The shRNA-mediated suppression of GADD153 attenuated NGLY1 knockdown-induced apoptosis in SK-MEL-2 cells. *Upper left panel:* The GFP/RFP-double positive cells indicated NGLY1-knockdown/control and NGLY1-knockdown/GADD153-knockdown cells. *Lower left panel:* The analysis of apoptotic (annexin V-stained) cells in the GFP/RFP-double positive cells. *Right panel:* The quantitative results of flow cytometry analysis in the cells with 72-hour induction of NGLY1-targeting shRNA. (**F**) The expression and activation of ER stress signaling-associated molecules detected by western blotting in SK-MEL-2 melanoma cells with indicated treatment. *Tun:* 2μM tunicamycin for 24 hours. *FL:* full-length. *CL:* cleavaged. *U:* unspliced. *S:* spliced. (G) The expression and activation of two eIF2α kinases, PKR (EIF2AK2) and PERK (EIF2AK3), detected by western blotting in SK-MEL-2 melanoma cells with indicated treatment. The All the quantitative data were presented as mean ± standard deviation (n=3) in the bar graphs (*P<0.05, t-test).

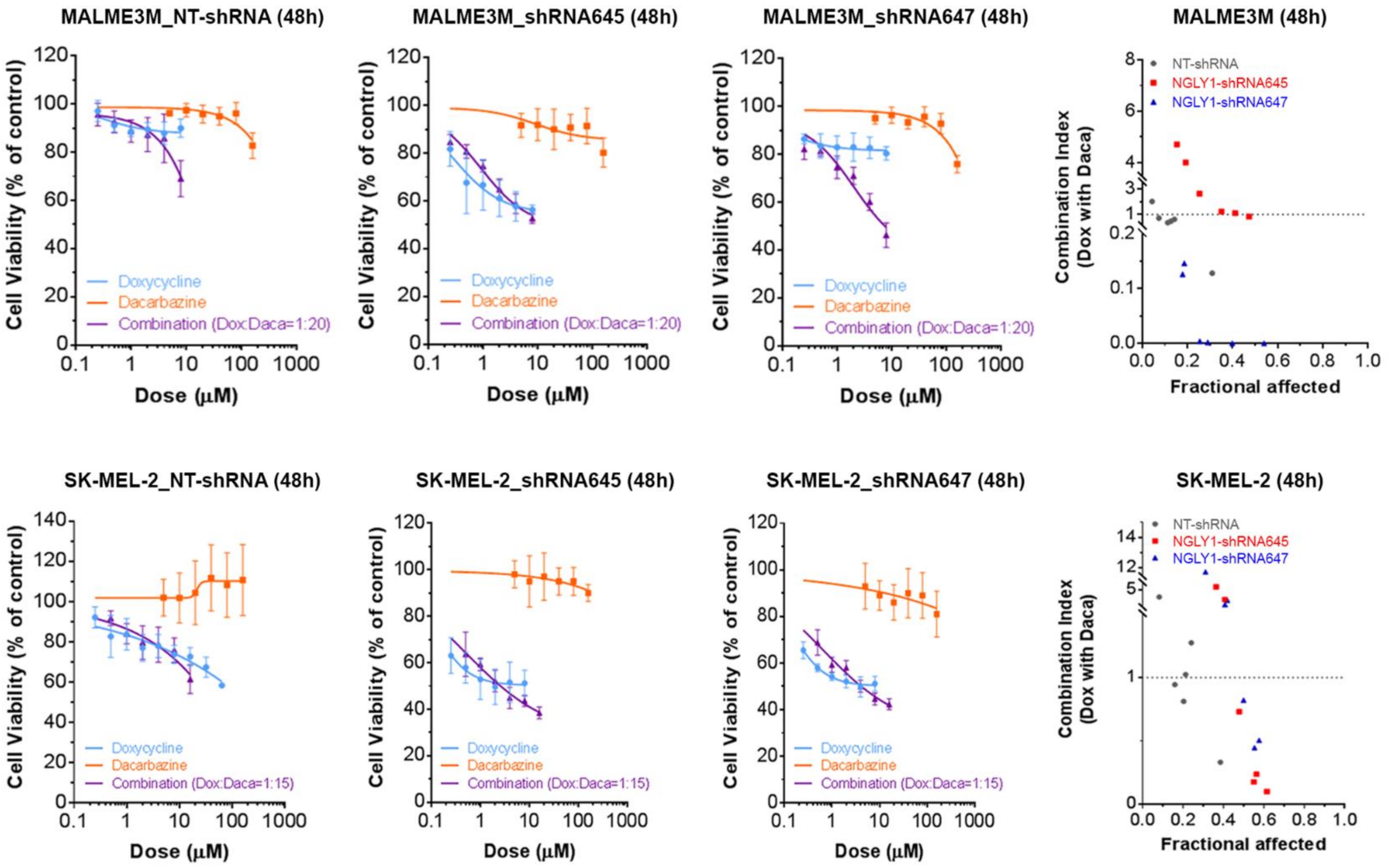

MALME3M_NT-shRNA (48h)
MALME3M_shRNA645 (48h)
MALME3M_shRNA647 (48h)
MALME3M (48h)
Cell Viability (% of control)
Dose (μM)
Doxycycline
Dacarbazine
Combination (Dox:Daca=1:20)
Combination Index (Dox with Daca)
Fractional affected
NT-shRNA
NGLY1-shRNA645
NGLY1-shRNA647
SK-MEL-2_NT-shRNA (48h)
SK-MEL-2_shRNA645 (48h)
SK-MEL-2_shRNA647 (48h)
SK-MEL-2 (48h)
Combination (Dox:Daca=1:15)

Supplementary Figure S3. The anticancer responses of the concomitant treatment of NGLY1 knockdown and dacarbazine for 48 hours in MALME3M and SK-MEL-2 melanoma cells. The calculation of combination indexes was performed using Calcusyn software. A combination index value <1 was considered synergistic. A combination index value <0.2 was considered highly synergistic. All cell viability data were presented as mean ± standard deviation (*n*=3).

Without doxycycline treatment

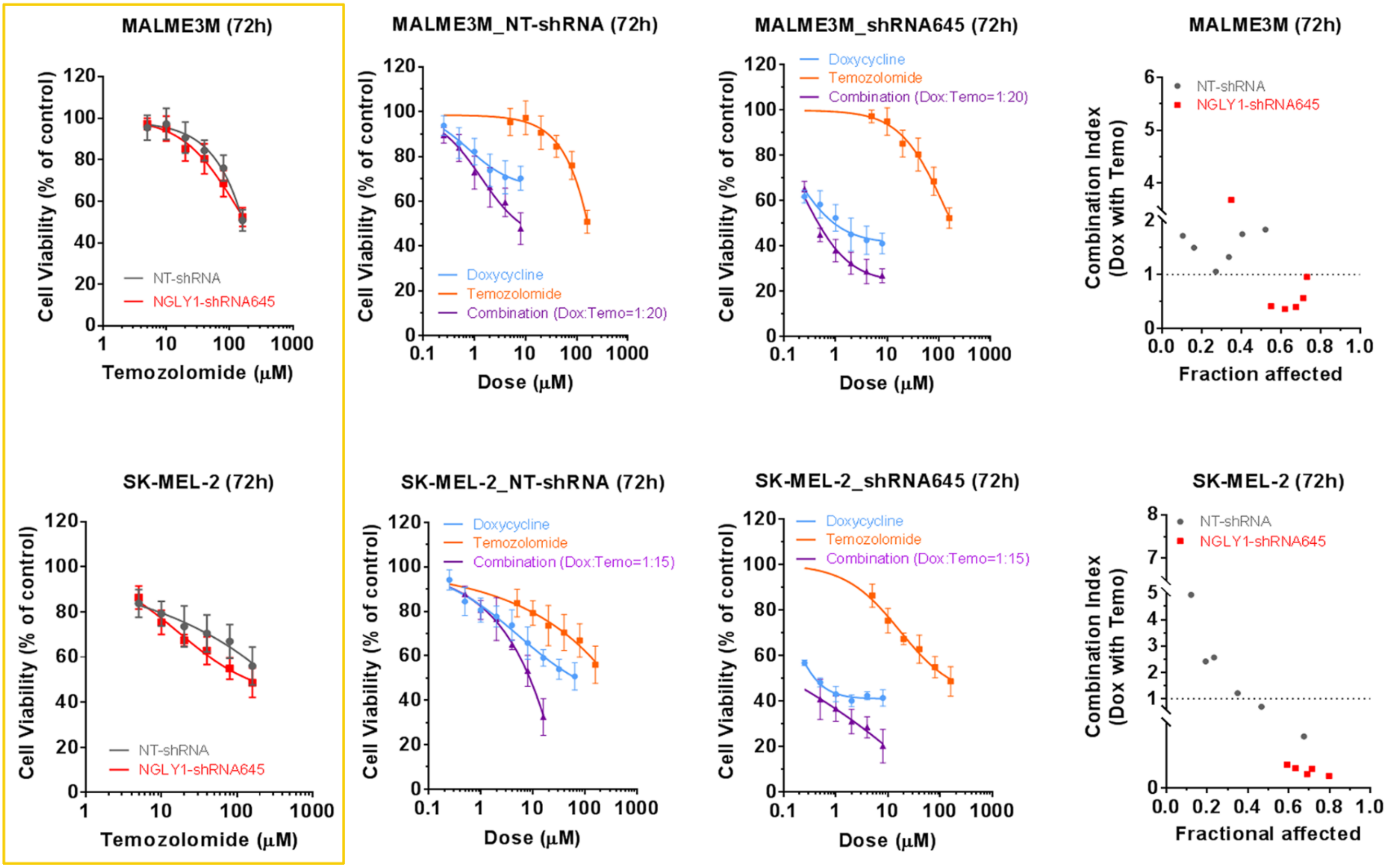

Supplementary Figure S4. The synergistic anticancer responses of NGLY1 knockdown and temozolomide treatment in MALME3M and SK-MEL-2 melanoma cells. The calculation of combination indexes was performed using Calcusyn software. A combination index value <1 was considered synergistic. A combination index value <0.2 was considered highly synergistic. All cell viability data were presented as mean ± standard deviation (*n*=3).

A

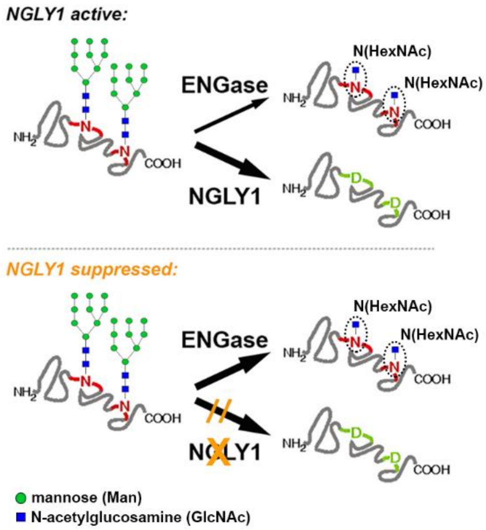

B

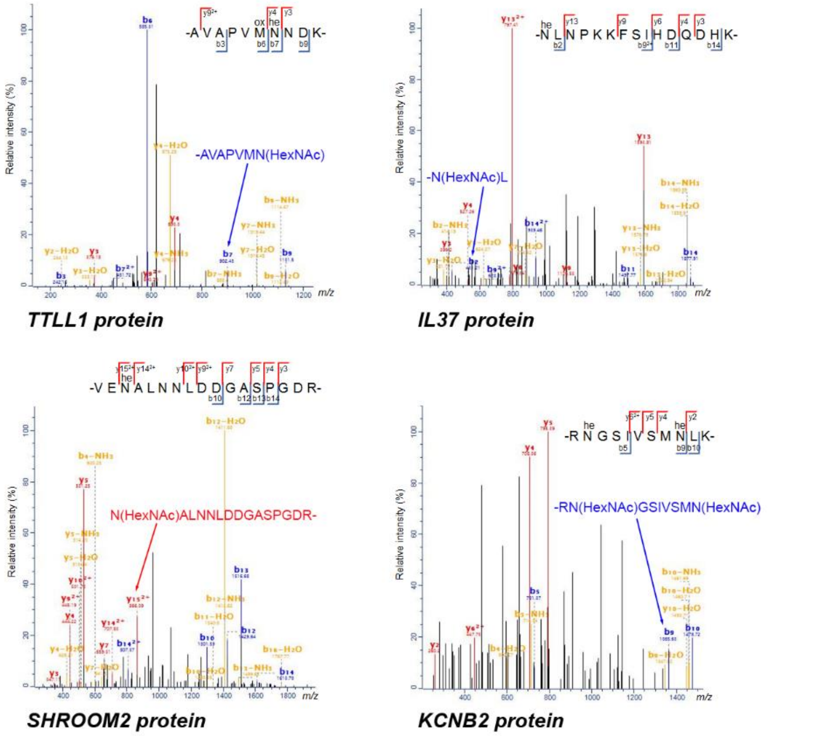

C

Mass spec information for GlcNAc modified peptides.

Modified peptide sequence [a]	Positions within proteins	UniProt accession number	Protein names	Localization prob. [b]	PEP [c]	Charge state	m/z	Mass error (ppm)	Score [d]
NLNPKKFSIHDQDHK	54	Q9NZH6	Interleukin-37 (IL37)	1.0	0.0292	2	1012.51343	-1.1	56.3
RNGSIVSMNLK	446	Q92953	Potassium voltage-gated channel subfamily B member 2 (KCNB2)	1.0	0.0342	2	812.914169	-0.9	40.2
RNGSIVSMNLK	453	Q92953	Potassium voltage-gated channel subfamily B member 2(KCNB2)	1.0	0.0342	2	812.914169	-0.9	40.2
AVAPVMNNDK	301	O95922	Probable tubulin polyglutamylase TTLL1 (TTLL1)	1.0	0.0209	2	639.305735	-1.1	76.2
VENALNNLDDGASPGDR	1514	Q13796	Protein Shroom2 (SHROOM2)	0.6	0.0123	2	980.448147	1.7	73.3

a: Underlined amino acid was GlcNAc modified
b: Localization probability calculated by MaxQuant. Value less than 0.75 was considered true positive
c: Posterior Error Probability (PEP_ of the identification, it is basically treated as p-value where less 0.05 was considered significant
d: Andromeda score for the best associated MS/MS spectrum, where higher is better.

D

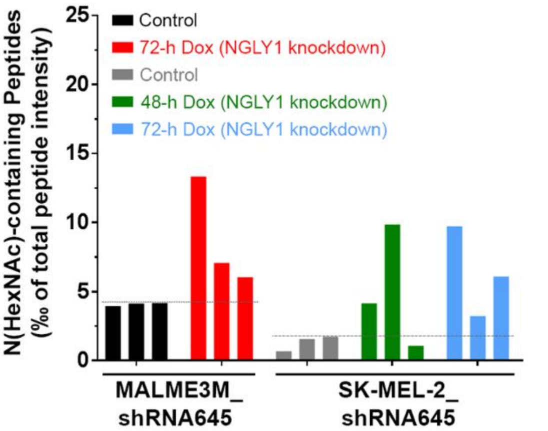

Supplementary Figure S5. Melanoma cells with the shRNA-mediated suppression of NGLY1 presented characteristic alterations in proteomics analysis. NGLY1 knockdown induced the increase of peptides containing GlcNAc-asparagine residues and additional perturbation in the proteomes of MALME3M and SK-MEL-2 cells with inducible shRNA targeting NGLY1. (**A**) Schematic illustration of enhanced ENGase-mediated formation of peptides containing GlcNAc-asparagine residues in the absence of NGLY1 in cells. (**B**) The MS/MS spectrum of tryptic peptide ions containing GlcNAc-asparagine residues annotated as N(HexNAc) of TTLL1, IL-37, SHROOM2, KCNB2 identified in the protein samples of MALME3M and SK-MEL2 cells with NGLY1 knockdown. (**C**) The mass spec information of representative GlcNAc-modified peptides. (**D**) The proportion of peptides containing GlcNAc-asparagine residues in the proteome of each cell sample was analyzed. The results of 3 biological replicates for each experimental setting were plotted. Prior to sample collection for analysis, 1μM doxycycline (dox) was used to treat cells for the indicated periods.

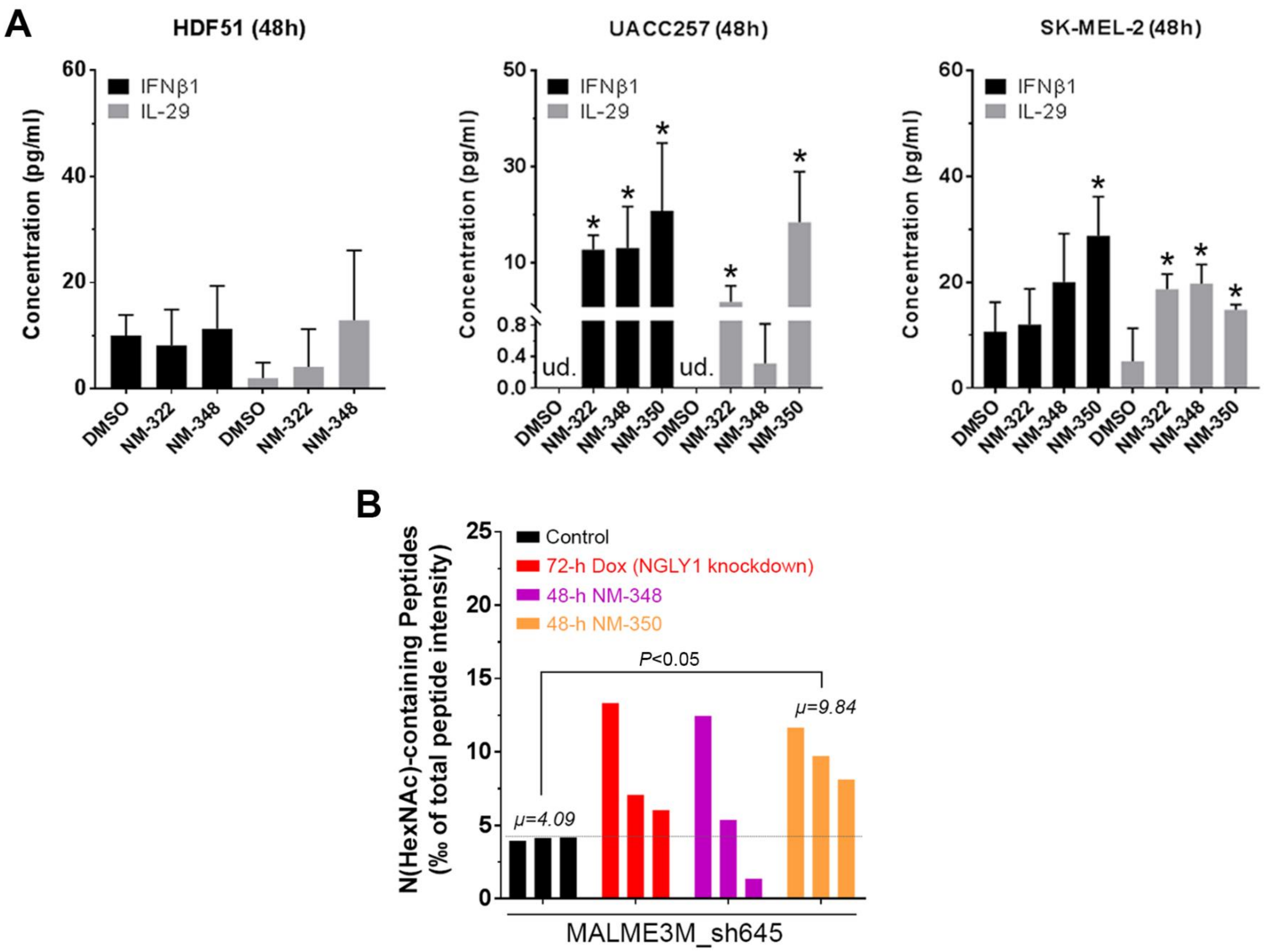

A
HDF51 (48h)
UACC257 (48h)
SK-MEL-2 (48h)
IFNβ1
IL-29
Concentration (pg/ml)
ud.
DMSO
NM-322
NM-348
NM-350
B
Control
72-h Dox (NGLY1 knockdown)
48-h NM-348
48-h NM-350
P<0.05
μ=9.84
μ=4.09
N(HexNAc)-containing Peptides
(‰ of total peptide intensity)
MALME3M_sh645

Supplementary Figure S6. The cytokine responses and glycopeptide features of melanoma cells treated with novel NGLY1 inhibitors. (**A**) The production of IFNβ1 and IL-29 was enhanced by novel small-molecule inhibitors targeting NGLY1. HDF51, UACC257, and SK-MEL-2 cells were treated using the indicated inhibitors. The conditional media of the cells were collected for cytokine analysis at the end of 48-hour drug treatment. The concentration of each inhibitor used in the test was 200µM. The data of cytokine analysis were presented as mean ± standard deviation (n=3,*P<0.05, t-test; ud., undetectable). (**B**) The proportion of peptides containing GlcNAc-asparagineresidues in the proteome of each cell sample was analyzed. The results of 3 biological replicates for each experimental setting were plotted. Prior to sample collection for analysis, 1µM doxycycline (dox), 200µM NM-348 or 200µM NM-350 was used to treat cells for the indicated periods.

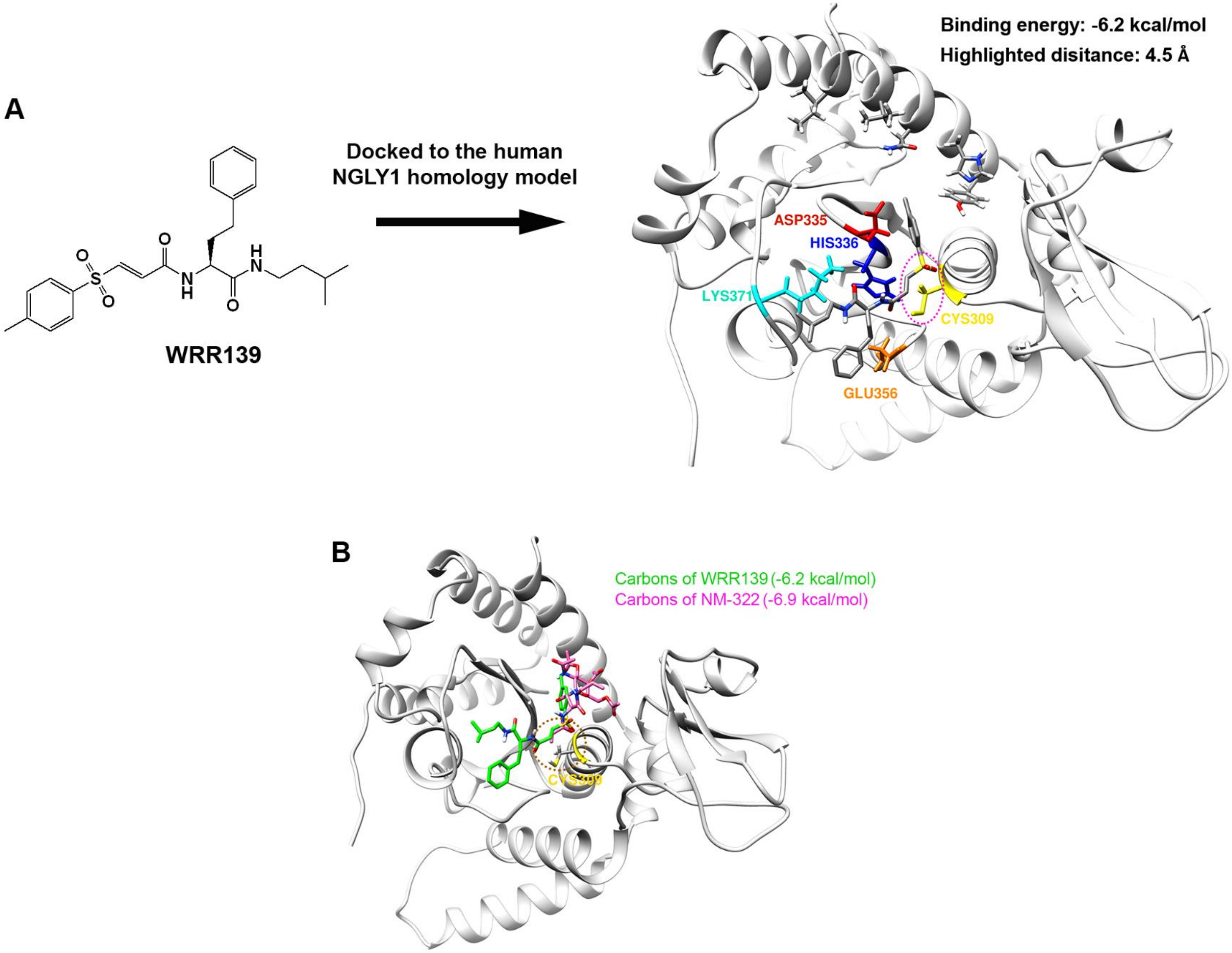
A
WRR139
Docked to the human NGLY1 homology model
Binding energy: -6.2 kcal/mol
Highlighted disitance: 4.5 Å
ASP335
HIS336
LYS371
CYS309
GLU356
B
Carbons of WRR139 (-6.2 kcal/mol)
Carbons of NM-322 (-6.9 kcal/mol)

Supplementary Figure S7. The binding poses of NGLY1 inhibitors WRR139 and NM-322 in the human NGLY1 homology model. (**A**) The most favorable binding pose of WRR139, a small molecule that has been shown to have NGLY1 inhibitory activity in cancer cells, in the homology model of human NGLY1 showed a good binding affinity with its electrophilic group pointed towards Cys309 in close proximity at the human NGLY1 catalytic site. (**B**) The distinct binding poses of WRR139 and NM-322 in the homology model of human NGLY1.

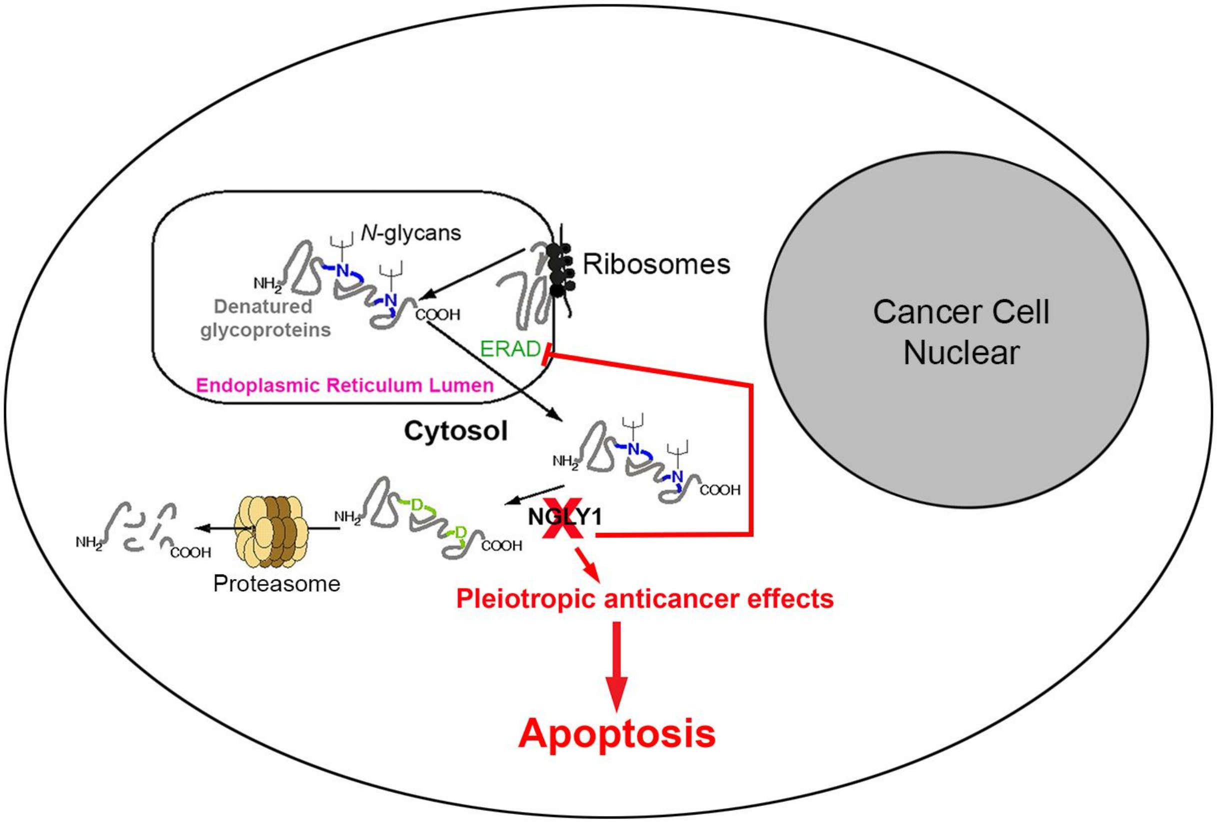
N-glycans
Ribosomes
NH2
Denatured glycoproteins
COOH
ERAD
Endoplasmic Reticulum Lumen
Cancer Cell Nuclear
Cytosol
NH2
COOH
NH2
COOH
NGLY1
NH2
COOH
Proteasome
Pleiotropic anticancer effects
Apoptosis

Supplementary Figure S8. The graphic summary of NGLY1 suppression-triggered anticancer responses in melanoma cells.

Supplementary Table S1. The list of cultured cells used in the study		
**Sample Name**	**Registry Name**[a]	**Note**[b]
**Human embryonic stem cells**		
WA09	WA09	Obtained from the WiCell Stem Cell Bank; feeder cell-free culture on Matrigel, passaged using L7 hPSC passaging solution
**Induced pluripotent stem cells from Human Dermal Fibroblasts (HDF)**		
NGLY1Pt1i-507	N/A	Sendai virus-mediated reprogramming in NGLY1-deficient patient's dermal fibroblasts (GM25990); feeder cell-free culture on Matrigel, passaged using L7 hPSC passaging solution
NGLY1Pt1i-508	N/A	Sendai virus-mediated reprogramming in NGLY1-deficient patient's dermal fibroblasts (GM25990); feeder cell-free culture on Matrigel, passaged using L7 hPSC passaging solution
NGLY1Pt1i-509	N/A	Sendai virus-mediated reprogramming in NGLY1-deficient patient's dermal fibroblasts (GM25990); feeder cell-free culture on Matrigel, passaged using L7 hPSC passaging solution
**Normal somatic cells**		
HDF51 (HDF-f)[c]	N/A	Human dermal fibroblasts, fetal skin; purchased from Sciencell
HM (HEMl)[c]	N/A	Human epidermal melanocytes (light), neonatal skin; purchased from Sciencell
HEMd	N/A	Human epidermal melanocytes (dark), neonatal skin; purchased from Sciencell
HDF418	N/A	Human dermal fibroblasts; isolated from the forearm skin biopsy sample of an adult male
**Cancer cells**		
UACC257	N/A	Human melanoma cells cultured using RPMI-1640 medium containing 10% FBS, enzymatic passaged using trypsin-EDTA
COLO829	N/A	Human melanoma cells cultured using RPMI-1640 medium containing 10% FBS, enzymatic passaged using trypsin-EDTA
SK-MEL-2	N/A	Human melanoma cells cultured using RPMI-1640 medium containing 10% FBS, enzymatic passaged using trypsin-EDTA
SK-MEL-5	N/A	Human melanoma cells cultured using RPMI-1640 medium containing 10% FBS, enzymatic passaged using trypsin-EDTA
451Lu	N/A	Human melanoma cells cultured using RPMI-1640 medium containing 10% FBS, enzymatic passaged using trypsin-EDTA
MEL1617	N/A	Human melanoma cells cultured using RPMI-1640 medium containing 10% FBS, enzymatic passaged using trypsin-EDTA
MALME3M	N/A	Human melanoma cells cultured using DMEM medium containing 10% FBS, enzymatic passaged using trypsin-EDTA
**Cells used for reprogramming**		
HDF (GM25990)	N/A	Human dermal fibroblasts derived from the skin biopsy sample of a patient with NGLY1 deficiency, cultured using DMEM medium containing 10% FBS, obtained from Coriell Biorepository

a. Name of cell line submitted to University of Massachusetts (UMass) International Stem Cell Registry
b. Somatic cell type, reprogramming method, culture condition, source of cells
c. Nomenclature used by the vendor

Supplementary Table S2. The list of primary antibodies and lectin used in the study		
**Antibody/Lectin Name**	**Catalog Number**	**Sources**
**Antibodies used in IHC or fluorescence staining**		
NGLY1	HPA036825	Millipore Sigma
TRA-1-81	09-0011	Stemgent
POU5F1	2840	Cell Signaling Technology
NANOG	MABD24	Millipore Sigma
TUBB3	MRB-435P	Biolegend (formerly Covance)
Smooth Muscle Actin (SMA)	MAB1420	R&D Systems
SOX17	AF1924	R&D Systems
DYKDDDDK Tag	MA1-142-A555	Thermo Fisher Scientific
Brachyury	sc-17745	Santa Cruz Biotechnology
**Antibodies used in immunoblotting**		
NGLY1	HPA036825	Millipore Sigma
pMEK1/2	9154	Cell Signaling Technology
MEK1/2	4694	Cell Signaling Technology
pERK1/2	4370	Cell Signaling Technology
ERK1/2	4696	Cell Signaling Technology
ACTIN	08691001	MP Biomedicals
POU5F1	2840	Cell Signaling Technology
NANOG	MABD24	Millipore Sigma
DYKDDDDK Tag	8146	Cell Signaling Technology
GADD153	NB600-1335	Novus Biologicals
pIRF3	4947	Cell Signaling Technology
IRF3	11904	Cell Signaling Technology
IRF7	13014	Cell Signaling Technology
pTBK1	5483	Cell Signaling Technology
TBK1	3504	Cell Signaling Technology
Ubiquitin	3936	Cell Signaling Technology
KDEL	ab12223	Abcam
ATF4	11815	Cell Signaling Technology
TCF11/NRF1	8052	Cell Signaling Technology
ZsGreen	632598	Takara
p-eIF2α	ab32157	Abcam
eIF2α	9722	Cell Signaling Technology
ATF6	65880	Cell Signaling Technology
XBP1	GTX102229	GeneTex
p-PKR	ab32036	Abcam
PKR	ab32506	Abcam
p-PERK	ab192591	Abcam
PERK	3192	Cell Signaling Technology
**Cytokine neuralization**		
IFNβ1	MAB814-100	R&D Systems
IL-29	MAB15981-100	R&D Systems
**Lectin used in fluorescence staining**		
UEA-I	FL-1061	Vector Laboratories

Supplementary Table S3. Immunohistochemistry staining intensity of NGLY1 in human normal skin and melanoma tissues

Pathology (# of Cases)	Negative (% of total cases)	Weak (% of total cases)[a]	Moderate (% of total cases)[a]	Strong (% of total cases)[a]
Normal skin or benign nevus (8)	8 (100)	0 (0)	0 (0)	0 (0)
Melanoma (33)[b]	19 (57.6)	7 (21.2)	6 (18.2)	1 (3.0)

[a] A tissue sample showing either weak, moderate and strong staining of NGLY1 is considered as NGLY1 positive. NGLY1-positive staining is associated with melanoma pathology (*P*=0.035, 2x2 contingency table, Fisher's exact test).

[b] The tumor sample from 1 of 36 melanoma patients was lost during the staining process. Since no visible cancer cell was found in the lymph node tissue samples supposed to contain metastatic melanoma cells of two patients, these two patients were excluded from analysis.

Supplementary Table S4. NGLY1 inactivation-induced alterations in protein abundance detected by proteomics analysis

Protein IDs	Genes	MALME3M Avg. % of total peptides (Control, *n*=3)	MALME3M Avg. % of total peptides (NGLY1-KD, *n*=3)	MALME3M Abundance fold change (KD/Control)*	SK-MEL-2 Avg. % of total peptides (Control, *n*=3)	SK-MEL-2 Avg. % of total peptides (NGLY1-KD, *n*=3)	SK-MEL-2 Abundance fold change (KD/Control)*
**Proteins with increased abundance commonly found in MALME3M and SK-MEL-2 cells with NGLY1 knockdown**							
P62937; Q9Y536	PPIA	1.3637	1.6296	1.1950	1.2622	4.4404	3.5180
P49903	SEPHS1	0.0000	0.0121	n.d. in control samples	0.0154	0.0397	2.5857
Q71U36; P68363; Q13748; P68366; Q6PEY2	TUBA1A; TUBA1B; TUBA3C; TUBA4A	1.9826	2.4477	1.2346	1.5769	3.7260	2.3628
P21796	VDAC1#	0.0696	0.1067	1.5331	0.1777	0.4163	2.3423
Q7KZF4	SND1	0.2903	0.3381	1.1646	0.2098	0.3684	1.7558
Q5VTE0; P68104; Q05639	EEF1A1P5; EEF1A1; EEF1A2	2.1861	2.6168	1.1970	2.0729	3.5754	1.7248
P25705	ATP5A1	0.3353	0.6229	1.8579	0.1367	0.2250	1.6451
P63261; P60709; P63267; P68133; P68032; P62736	ACTG1; ACTB; ACTG2; ACTA1; ACTC1; ACTA2	15.8514	19.9151	1.2564	12.4899	20.5437	1.6448
P30101	PDIA3	0.8989	1.4247	1.5849	0.4545	0.7444	1.6379
P10809	HSPD1#	1.9390	2.3958	1.2356	1.8716	2.8825	1.5401
O43707	ACTN4	0.3716	0.5029	1.3533	0.4010	0.6119	1.5258
P50991	CCT4	0.2826	0.4360	1.5428	0.3104	0.4672	1.5052
P49006	MARCKSL1	0.5431	0.6807	1.2533	0.4021	0.4889	1.2160
Q8NC51	SERBP1	0.3675	0.5570	1.5155	0.4059	0.4819	1.1871
Q15942	ZYX	0.0284	0.1168	4.1198	0.0282	0.0322	1.1419
Q96QR8	PURB	0.0230	0.0302	1.3177	0.0370	0.0411	1.1104
**Proteins with reduced abundance commonly found in MALME3M and SK-MEL-2 cells with NGLY1 knockdown**							
P14314	PRKCSH	0.1371	0.0000	n.d. in NGLY1-KD samples	0.5048	0.4438	0.8792
P08670	VIM#	9.4669	6.9682	0.7361	6.3892	4.9337	0.7722
P61978	HNRNPK	0.3374	0.2840	0.8418	0.4715	0.3634	0.7706
P16949	STMN1	0.1663	0.0000	n.d. in NGLY1-KD samples	0.2708	0.2026	0.7482
P55072	VCP#	0.2390	0.1532	0.6412	0.2363	0.1755	0.7428
Q99714	HSD17B10	0.0246	0.0000	n.d. in NGLY1-KD samples	0.0513	0.0331	0.6443
P11021	HSPA5 (GRP78)	0.4052	0.2222	0.5485	0.5763	0.3493	0.6062
P14625; Q58FF3	HSP90B1 (GRP94)	0.6381	0.2500	0.3918	0.2317	0.1370	0.5914
P35268	RPL22	0.3121	0.2614	0.8377	0.4938	0.2506	0.5075
Q5JTV8	TOR1AIP1	0.0220	0.0000	n.d. in NGLY1-KD samples	0.0031	0.0000	n.d. in NGLY1-KD samples
P34932	HSPA4	0.0063	0.0000	n.d. in NGLY1-KD samples	0.0063	0.0000	n.d. in NGLY1-KD samples
P52907	CAPZA1	0.0123	0.0000	n.d. in NGLY1-KD samples	0.0109	0.0000	n.d. in NGLY1-KD samples
Q01082	SPTBN1	0.0369	0.0000	n.d. in NGLY1-KD samples	0.0115	0.0000	n.d. in NGLY1-KD samples
Q14847	LASP1	0.0313	0.0000	n.d. in NGLY1-KD samples	0.0198	0.0000	n.d. in NGLY1-KD samples
P40925	MDH1	0.1165	0.0000	n.d. in NGLY1-KD samples	0.0219	0.0000	n.d. in NGLY1-KD samples
P46779	RPL28	0.0293	0.0000	n.d. in NGLY1-KD samples	0.0296	0.0000	n.d. in NGLY1-KD samples
P13667	PDIA4	0.2196	0.0000	n.d. in NGLY1-KD samples	0.0362	0.0000	n.d. in NGLY1-KD samples
P35527; CON__P35527	KRT9	0.0783	0.0000	n.d. in NGLY1-KD samples	0.0640	0.0000	n.d. in NGLY1-KD samples
P06454	PTMA	0.0983	0.0442	0.4497	0.0767	0.0000	n.d. in NGLY1-KD samples
P62829	RPL23	0.0742	0.0000	n.d. in NGLY1-KD samples	0.1392	0.0000	n.d. in NGLY1-KD samples
P62805	HIST1H4A (histone H4)	1.2159	0.2125	0.1748	0.4122	0.0000	n.d. in NGLY1-KD samples

Genes highlighted in red showed increased abundance in SK-MEL-2 cells with the 48-hour treatment of 200μM NM-350, in comparison with control cells.

Genes highlighted in blue showed reduced abundance in SK-MEL-2 cells with the 48-hour treatment of 200μM NM-350, in comparison with control cells.

* n.d., non-detectable.

Less than 10% of abundance fold changes in SK-MEL-2 cells with the 48-hour treatment of 200μM NM-350, in comparison with control cells

Supplementary Table S5. Differentially expressed genes (*P*<0.05 & Average fold change ≥2) in human melanoma cells with NGLY1 knockdown

gene probe #	Gene name[a]	Average fold change (log2)[b]	*P* value
13	ATF3	1.568	0.0016611
36	AXUD1	1.252	0.0004843
57	BEX2	1.112	0.0114698
51	BIRC3	1.132	0.0198327
6	CCL5	2.125	0.0047061
4	CCL5	2.369	0.0012786
74	CDCA7	-1.035	0.0046373
68	CDKN2C	1.058	0.0276974
44	CENTA1	1.162	0.0010219
53	CFB	1.122	0.0392379
78	CRYAB	-1.015	0.0098900
65	DDIT3	1.062	0.0105068
56	DDX58	1.113	0.0008328
25	DHX58	1.354	0.0000114
28	EGR1	1.328	0.0001190
38	EGR2	1.223	0.0004354
12	EPSTI1	1.584	0.0010029
48	FABP7	-1.133	0.0022050
84	FOS	0.999	0.0013192
59	GAPDHS	-1.101	0.0327541
37	GBP1	1.249	0.0014629
63	GPM6B	-1.072	0.0100371
1	HCP5	2.630	0.0015499
52	HERC5	1.127	0.0000739
8	HLA-B	1.951	0.0002007
30	HLA-C	1.303	0.0020300
61	HLA-F	1.098	0.0114828
15	HLA-F	1.491	0.0000087
43	HMGCL	1.174	0.0001207
35	IFI44	1.261	0.0000125
31	IFI44L	1.277	0.0024527
18	IFIH1	1.478	0.0000116
49	IFIT1	1.133	0.0005623
29	IFIT1	1.313	0.0007546
7	IFIT2	1.997	0.0010418
10	IFIT3	1.724	0.0001275
9	IFIT3	1.922	0.0000341
23	IFITM1	1.407	0.0037163
2	IFNB1	2.436	0.0017186
27	IL29	1.335	0.0054003
83	IL8	1.002	0.0422249
33	IRF1	1.272	0.0039862
85	IRF7	0.996	0.0004498
46	IRF7	1.154	0.0003181
34	ISG15	1.263	0.0003385
21	KLF4	1.428	0.0013494
41	LOC100008588	-1.177	0.0371693
19	LOC100132564	1.475	0.0073257
45	LOC100133565	-1.154	0.0022528
17	NGLY1	-1.481	0.0000005
26	OAS1	1.338	0.0000529
22	OAS1	1.421	0.0000192
70	OAS2	1.045	0.0001903
54	OAS2	1.119	0.0003339
67	OAS3	1.060	0.0001514
16	OASL	1.485	0.0005257
5	OASL	2.274	0.0000435
76	PARP12	1.023	0.0079003
58	PARP9	1.101	0.0000546
11	PMAIP1	1.660	0.0006323
39	PSMB9	1.213	0.0049945
24	PTGS2	1.394	0.0099303
77	RARRES3	1.022	0.0000657
32	RN5S9	1.274	0.0325331
3	RSAD2	2.411	0.0000004
71	RTP4	1.045	0.0002050
62	SAMD9	1.078	0.0005239
40	SAMD9L	1.204	0.0020208
81	SEMA5A	-1.006	0.0287923
55	SERTAD1	1.117	0.0013974
14	SLC15A3	1.500	0.0004396
86	SP110	0.996	0.0000227
69	SP110	1.050	0.0000635
42	SP110	1.175	0.0000145
72	TAP1	1.038	0.0001089
64	TMEM140	1.068	0.0029536
73	TNFRSF12A	1.037	0.0453292
66	TNFSF10	1.061	0.0145028
50	TRIM22	1.133	0.0020824
47	USP18	1.150	0.0001262
75	XAF1	1.033	0.0028008
20	XAF1	1.447	0.0000637
79	ZC3HAV1	1.015	0.0002501
60	ZC3HAV1	1.100	0.0000965
80	ZNFX1	1.011	0.0001059

[a] The upregulation of genes with gray shading was observed in human melanoma cells in response to IL-29 treatment (Guenterberg, *et al.*, 2010). The expression of genes highlighted in red previously have been liked to anticancer activity (*e.g.*, cell cycle arrest, apoptosis, or mobility suppression in cancer cells), while the expression of genes highlighted in blue have been associated with the survival, proliferation and invasiveness of cancer cells or with a poor prognosis in melanoma patients.

[b] Positive values indicate expression changes in upregulation. Negative values indicate expression changes in downregulation.

References cited in Supplementary Materials and Methods

1 Cox J, Neuhauser N, Michalski A, Scheltema RA, Olsen JV, Mann M. Andromeda: a peptide search engine integrated into the MaxQuant environment. *J Proteome Res* 2011; **10**:1794-1805.

2 Dang C-H, Nguyen C-H, Nguyen T-D, Im C. Synthesis and characterization of N-acyl-tetra-O-acyl glucosamine derivatives. *RSC Adv* 2014; **4**:6239-6245.

3 Greig IR, Macauley MS, Williams IH, Vocadlo DJ. Probing synergy between two catalytic strategies in the glycoside hydrolase O-GlcNAcase using multiple linear free energy relationships. *Journal of the American Chemical Society* 2009; **131**:13415-13422.

4 Premdjee B, Adams AL, Macmillan D. Native N-glycopeptide thioester synthesis through N-->S acyl transfer. *Bioorganic & Medicinal Chemistry Letters* 2011; **21**:4973-4975.

5 Tropper FD, Andersson FO, Braun S, Roy R. Phase Transfer Catalysis as a General and Stereoselective Entry into Glycosyl Azides from Glycosyl Halides. *Synthesis* 1992:618-620.

6 Biasini M, Bienert S, Waterhouse A *et al.* SWISS-MODEL: modelling protein tertiary and quaternary structure using evolutionary information. *Nucleic Acids Research* 2014.

7 Morris GM, Huey R, Lindstrom W *et al.* AutoDock4 and AutoDockTools4: Automated docking with selective receptor flexibility. *Journal of Computational Chemistry* 2009; **30**:2785-2791.

8 Trott O, Olson AJ. AutoDock Vina: improving the speed and accuracy of docking with a new scoring function, efficient optimization, and multithreading. *Journal of Computational Chemistry* 2010; **31**:455-461.
